# BS-virus-finder: virus integration calling using bisulfite sequencing data

**DOI:** 10.1093/gigascience/gix123

**Published:** 2017-12-18

**Authors:** Shengjie Gao, Xuesong Hu, Fengping Xu, Changduo Gao, Kai Xiong, Xiao Zhao, Haixiao Chen, Shancen Zhao, Mengyao Wang, Dongke Fu, Xiaohui Zhao, Jie Bai, Likai Mao, Bo Li, Song Wu, Jian Wang, Shengbin Li, Huangming Yang, Lars Bolund, Christian N S Pedersen

**Affiliations:** Bioinformatics Research Center, Aarhus University, C. F. Møllers Allé 8, DK-8000, Aarhus C, Denmark; Forensics Genomics International (FGI), BGI-Shenzhen, BeiShan Industrial Zone, Yantian District, Shenzhen, Guangdong 518083, China; BGI-Shenzhen, BeiShan Industrial Zone, Yantian District, Shenzhen, Guangdong 518083, China; College of Computer Science and Technology, Qingdao University, Qingdao 266071, China; Department of Veterinary Clinical and Animal Sciences, University of Copenhagen, Grønnegårdsvej 15, DK-1870 Frederiksberg C, Denmark; College of Mathematics & Statistics, Changsha University of Science and Technology, Changsha 410114, China; James D. Watson Institute of Genome Sciences, Hangzhou 310058, China; The Affiliated Luohu Hospital of Shenzhen University, Shenzhen University, Shenzhen 518000, China; Department of Biomedicine, Aarhus University, Vennelyst Boulevard 4, DK-8000 Aarhus C, Denmark; Department of Biology, University of Copenhagen, Ole Maaløes Vej 5, DK-2200 Copenhagen N, Denmark; BGI Education Center, University of Chinese Academy of Sciences, Beijing 100049, China; Shenzhen Key Laboratory of Forensics, BGI-Shenzhen, Shenzhen 518083, China; China National GeneBank, BGI-Shenzhen, Shenzhen 518120, China; College of Medicine and Forensics, Xi'an Jiaotong University, Xi'an 710049, China

**Keywords:** bisulfite sequencing, carcinogenesis, virus integration

## Abstract

**Background:**

DNA methylation plays a key role in the regulation of gene expression and carcinogenesis. Bisulfite sequencing studies mainly focus on calling single nucleotide polymorphism, different methylation region, and find allele-specific DNA methylation. Until now, only a few software tools have focused on virus integration using bisulfite sequencing data.

**Findings:**

We have developed a new and easy-to-use software tool, named BS-virus-finder (BSVF, RRID:SCR_015727), to detect viral integration breakpoints in whole human genomes. The tool is hosted at https://github.com/BGI-SZ/BSVF.

**Conclusions:**

BS-virus-finder demonstrates high sensitivity and specificity. It is useful in epigenetic studies and to reveal the relationship between viral integration and DNA methylation. BS-virus-finder is the first software tool to detect virus integration loci by using bisulfite sequencing data.

## Introduction

**Figure 1:**
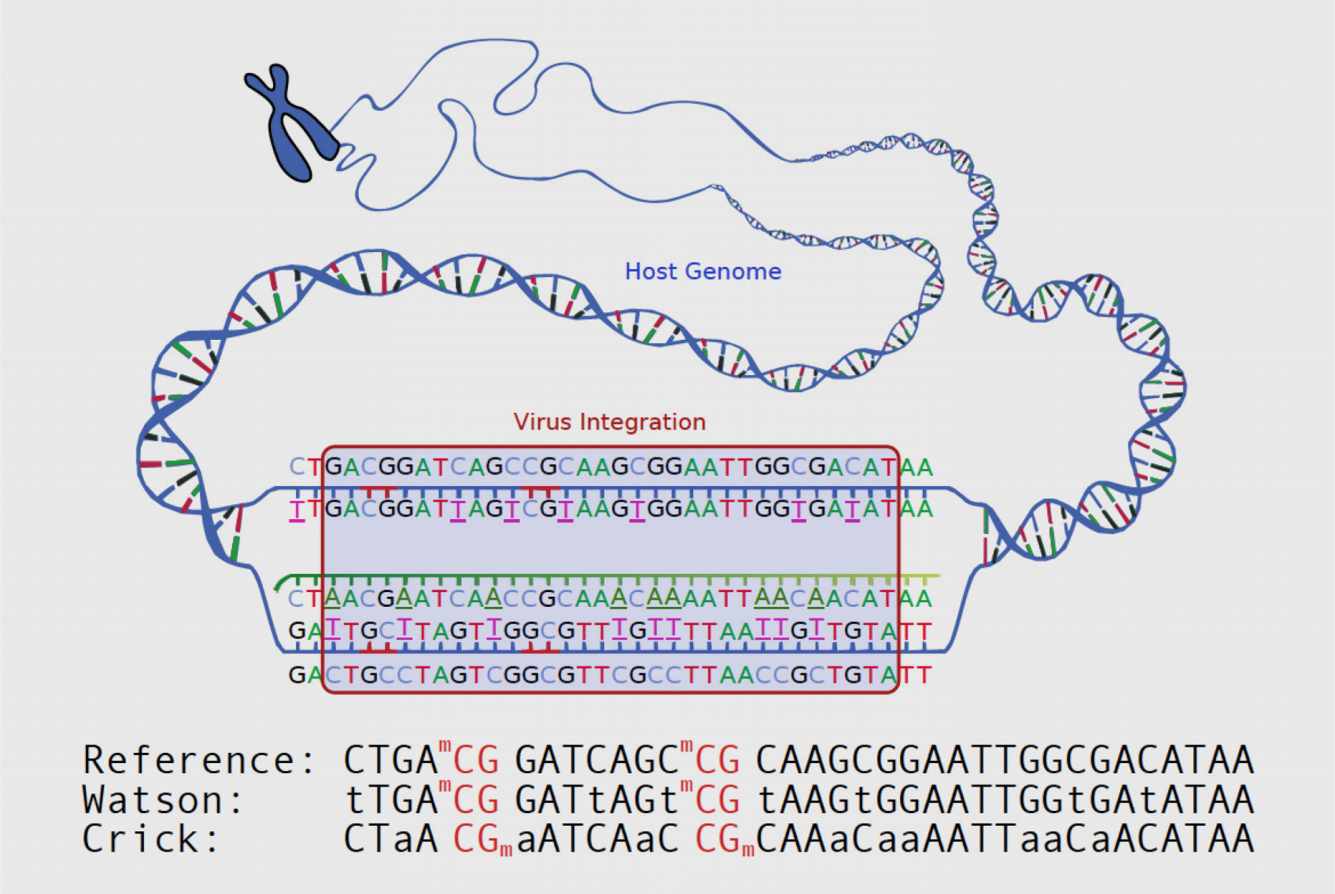
The illustration of bisulfite-altered sequence to the original.

DNA methylation plays a crucial role in many areas including development [1, 2] and X chromosome inactivation [3] by regulating genetic imprinting and epigenetic modification without altering DNA sequences. Previous studies have shown a strong association of DNA methylation with cancer. The methylation status altering related to carcinogenesis [4], cancer recurrence [5], and metastasis [6] has already been revealed by emerging bisulfite sequencing (BS) technology. BS technology can investigate DNA methylation changes with single-base accuracy. Treatment of DNA with bisulfite converts unmethylated cytosine residues to uracil, but leaves 5-methylcytosine residues unmodified [7]. Thus, bisulfite treatment introduces specific changes in the DNA sequence that depend on the methylation status of individual cytosine residues, yielding single-nucleotide resolution information about the methylation status of a segment of DNA (Fig. 1). Various analyses can be performed on the altered sequences to retrieve this information. BS technology can reveal differences between cytosines and thymidine and sequence change resulting from bisulfite conversion. For the bases without methylation, all Cs will change to Ts on both strands. After directional library preparation, we have 2 different conversions: the Watson and the Crick strands, as shown in Fig. 1. On the Watson strand, methylated C remains C, and unmethylated C changes to T. On the Crick strand, the reverse complement happens; i.e., methylated C remains C, but in sequenced reads it is reverse-complemented to G, and unmethylated C changes to T, leading to the reverse-complement base A in sequenced reads. As base C can either be methylated or unmethylated, we can use International Union of Pure and Applied Chemistry (IUPAC) nucleotide codes “Y” and “R” to represent C/T and G/A, respectively. So, after bisulfite treatment, base C changes to Y on the Watson strand, and base G changes to R on the Crick strand.

Whole-genome-based bisulfite sequencing (WGBS) has been developed to detect DNA methylation. Recent clinical studies showed that DNA methylation is associated with viral integration [8, 9]. Whole-genome BS data can be analyzed to investigate the sequence mapping and alignment via BSMAP [10], Bismark [11], and bwa-meth [12], to detect different methylation regions (DMRs) via the software QDMR [13], DMAP [14], and SMAP [15], to identify single nucleotide polymorphisms (SNPs) via BS-SNPer [16] and Bis-SNP [17], and to find allele-specific DNA methylation via SMAP [15] and Methy-Pipe [18]. However, none of them can be used for virus integration loci calling, and no software tool is currently available to detect virus integration loci by analyzing BS data. Therefore, we have developed a software tool to detect the virus integration loci by genome-wide BS analysis.

### Description of *in silico* and real data

**Figure 2:**
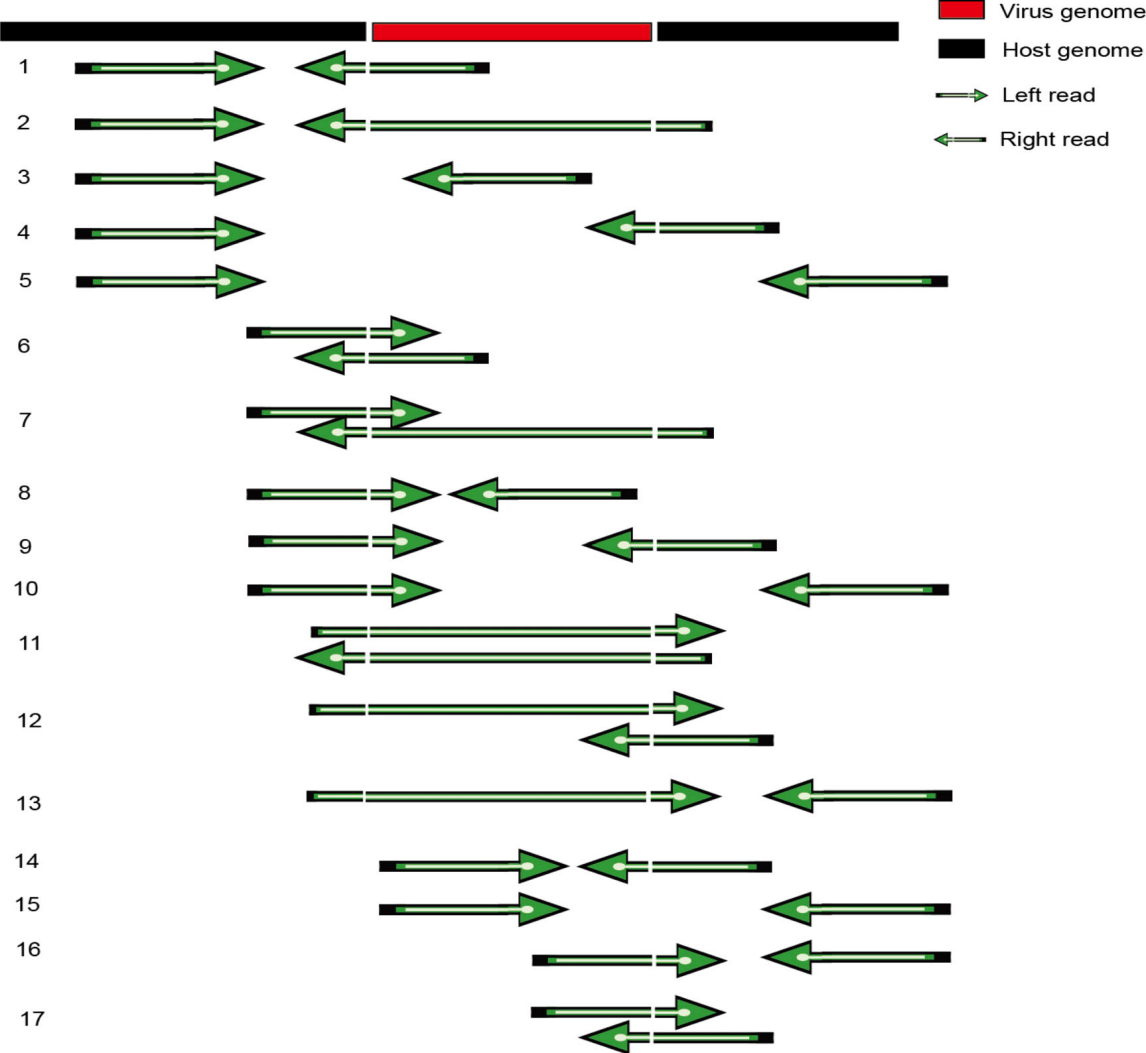
Principal types of mapping reads around the viral integration site.

Different types of paired-end (PE) reads (50 base pairs [bp], 90 bp, 150 bp) that include 700 breakpoints in chromosome 1 (chr 1) of GRCh38 were simulated in our study. Input fragments of 50 to 400 bp were randomly selected from chr 1 in the GRCh38 assembly of the human genome. The hepatitis B virus (HBV) genome (GenBank: X04615.1) was used in our simulation. Its integration length was between 45 bp and 180 bp. We cut HBV-containing segments with given PE insert size at all possible positions on every integration event. After alignment, mapping accuracy of each of the 17 different types of read mappings was calculated (Fig. 2). Mapping accuracy varied among the 17 types of read mappings in our simulation (Figs S1, S2, S3). In summary, the accuracies of several kinds of the read mappings were low (Tables S1, S2, S3), which may raise the false-negative rate. Generally, however, bwa-meth [12] performed very well.

Bisulfite sequencing is a sophisticated technique to study DNA cytosine methylation. Bisulfite treatment followed by polymerase chain reaction (PCR) amplification specifically converts unmethylated cytosine to thymine. By cooperating with next-generation sequencing technology, it is able to detect the methylation status of every cytosine in the whole genome. Moreover, longer reads make it possible to achieve higher accuracy. Besides simulated data, the PLC/PRF/5 hepatocellular carcinoma cell lines (from American Type Culture Collection [ATCC], Manassas, VA, USA) were cultured as previously described [19]. The cell line was validated by STR makers (Fig. S4). We performed whole-genome sequencing (WGS) and WGBS sequencing of this cell line (the results are shown in Table S4). Table 1 shows the analysis result for WGS data, which were compared with the output results analyzed by Vy-per [20], virus-clip [21], and Virus Finder2 [22].

**Table 1:** The comparison of BS-virus-finder with other software using real data

	BSVF			Vy-per			Virus-clip			Virus Finder2		
Chr	HB	VB	VE	HB	VB	VE	HB	VB	VE	HB	VB	VE
chr1	143 272 758	2945	3102									
chr2	-			-			52 018 758	207	281			
chr3^*^	131 451 702	1212	1322	-			131 451 701	1282	1403	131 451 701	1405	
chr3^*^	131 453 124	1416	1515	-			131 453 353	1416	1538			
chr4^*^	180 586 417	136	378							180 586 416	59	
chr4^*^	180 587 608	394	594	180 586 607	167	231	180 587 608	500	632	180 587 607	634	
chr5^*^	1 297 478	1174	1315	-			1 297 478	1241	1385	1 297 477	1388	
chr7	110 894 616	2739	2748									
chr8^*^	35 446 380	2389	2459	35 446 214	2402	2455	35 446 601	2390	2519	35 446 392	2396	2608
chr8	-						106 944 290	698	1077			
chr11^*^	65 040 943	2631	2767	-			-			65 040 964	2532	
chr12^*^	109 573 899	721	815	109 573 677	668	734	109 573 899	705	815			
chr13	33 088 123	1521	1603	**-**			**-**					
chr13	33 088 561	1917	2066	-			33 088 561	1995	2133	33 088 560	2133	
chr16^*^	69 947 046	2055	2826									
chr16^*^	70 169 959	2055	2735							70 169 971	2064	2240
chr16	74 425 602	2062	2665									
chr17^*^	82 105 786	407	489	82 105 984	368	435	82 105 783	347	489			
chr17^*^	82 107 626	2177	2321	-			82 107 710	2048	2159	82 107 625	2045	
chr19	41 783 064	687	804	-			41 782 971	761	905			
chr20	20 473 566	2415	2565									

BSVF used WGBS data, and other software used WGS data.

^*^Supported by previous fluorescence in situ hybridization (FISH) experiments [8].

Abbreviations: HB: host breakpoint;

VB: virus begin is the revealed leftmost position on virus;

VE: virus end is the rightmost position on virus.

## Methods

### Sample preparation

PLC/PRF/5 hepatocellular carcinoma cell line was obtained from ATCC (Manassas, VA, USA) and was cultured as previously described [19] and validated by STR makers (Fig. S4). In total, 15 μg of DNA was extracted to perform WGS and WGBS sequencing. Sample concentration was detected by fluorometer (QubitFluorometer, Invitrogen). Sample integrity and purification was determined by Agarose Gel Electrophoresis.

### Whole-genome sequencing

About 1.5 μg of gDNA was sonicated to 100–300-bp fragment genome DNA by Sonication (Covaris) and purified with QIAquick PCR Purification Kit (Qiagen). Adapter ligation and target insert size fragment recovering and quantifying library by real-time quantitative PCR (QPCR; TaqMan Probe) were then performed. The qualified library was sequenced on an Illumina Hiseq X Ten platform, and 150 bp of PE reads were obtained. In total, around 90 G of clean data were generated.

### Whole-genome bisulfite sequencing

About 3 μg of gDNA were sonicated to 100–300 bp by Sonication (Covaris) and purified with MiniElute PCR Purification Kit (QIAGEN). A single “A” nucleotide was added to the 3^΄^ ends of the blunt fragments. Methylated adapters were then purified and added to the 5^΄^ and 3^΄^ ends of each strand in the genomic fragment. Sizes 300–400 bp were selected. DNA was then purified with QIAquick Gel Extraction Kit (QIAGEN) and bisulfite treated with Methylation-Gold Kit (ZYMO). Finally, PCR was conducted and sizes 350–400 bp were selected and purified with QIAquick Gel Extraction kit (QIAGEN). Qualified library was amplified on cBot to generate the cluster on the flowcells (TruSeq PE Cluster Kit V3–cBot–HS, Illumina). The flowcells were sequenced for 150 bp of PE reads on the HiSeq X Ten platform, and more than 90G of clean data were generated.

## Data analysis

The read coverage situation for 1 integration is shown in Fig. 3. Four steps were implemented to detect virus integration:

1. Alignment: We use bwa-meth [12] to align bisulfite-treated sequencing reads to a hybrid reference that contains both human genome and virus sequences. For chimeric reads from the junction parts, BWA-MEM [23] will align it to 1 organism and mark the unmapped part as soft clipping, which is in fact from the other organism. This enables us to find breakpoints directly from the alignment.
2. Clustering: After alignment, the result was filtered. We select read pairs with 1 read match by the following criterion: The Phred-scaled mapping quality is larger than 30 (≥30), and at least 1 soft clipping is longer than 5 bp (≥5). The mapped parts of reads, which is marked as “M” by its CIGAR string, cover the human reference genome. For paired reads, we also add the gap between 2 mapped reads to their covered region, making read 1 and read 2 continuously covered on the human reference. Each continuous region with at least 1 bp of overlap is defined as a cluster. All reads involved are selected to form the cluster. The remaining soft clippings are viral junction candidates. Read pairs with 1 read mapped on the virus also indicate a potential virus junction between the read pairs.
3. Assembling: Within 1 cluster, all soft clipping start sites are collected. The position with the most abundance of start sites is identified as the most likely candidate breakpoint. All clipping sequences in the cluster are extracted and aligned together. A restore algorithm was used to calculate the most possible base in each position based on the aligned bases and their sequencing quality. The algorithm is based on a Bayesian model, where we compute the *posteriori* probability estimation for A, C, G, T as:
  (1) \begin{equation*} \begin{array}{@{}l@{}} \displaystyle P({T_i}|D) = \frac{{P({T_{Wi}})P(D|{T_{Wi}})}}{{\sum\limits_{x = 1}^s {P({T_{Wx}})P(D|{T_{Wx}})} }} \times \frac{{P({T_{Ci}})P(D|{T_{Ci}})}}{{\sum\limits_{x = 1}^s {P({T_{Cx}})P(D|{T_{Cx}})} }}\nonumber\\ \displaystyle \quad \quad \quad \quad = {C_0} \times P(D|{T_{Wi}}) \times P(D|{T_{Ci}})\nonumber\\ \displaystyle {C_0} = \frac{{P({T_{Wi}})}}{{\sum\limits_{x = 1}^S {P({T_{Wx}})P(D|{T_{Wx}})} }} \times \frac{{P({T_{Ci}})}}{{\sum\limits_{x = 1}^S {P({T_{Cx}})} P(D|{T_{Cx}})}} \end{array}\end{equation*}

**Figure 3:**
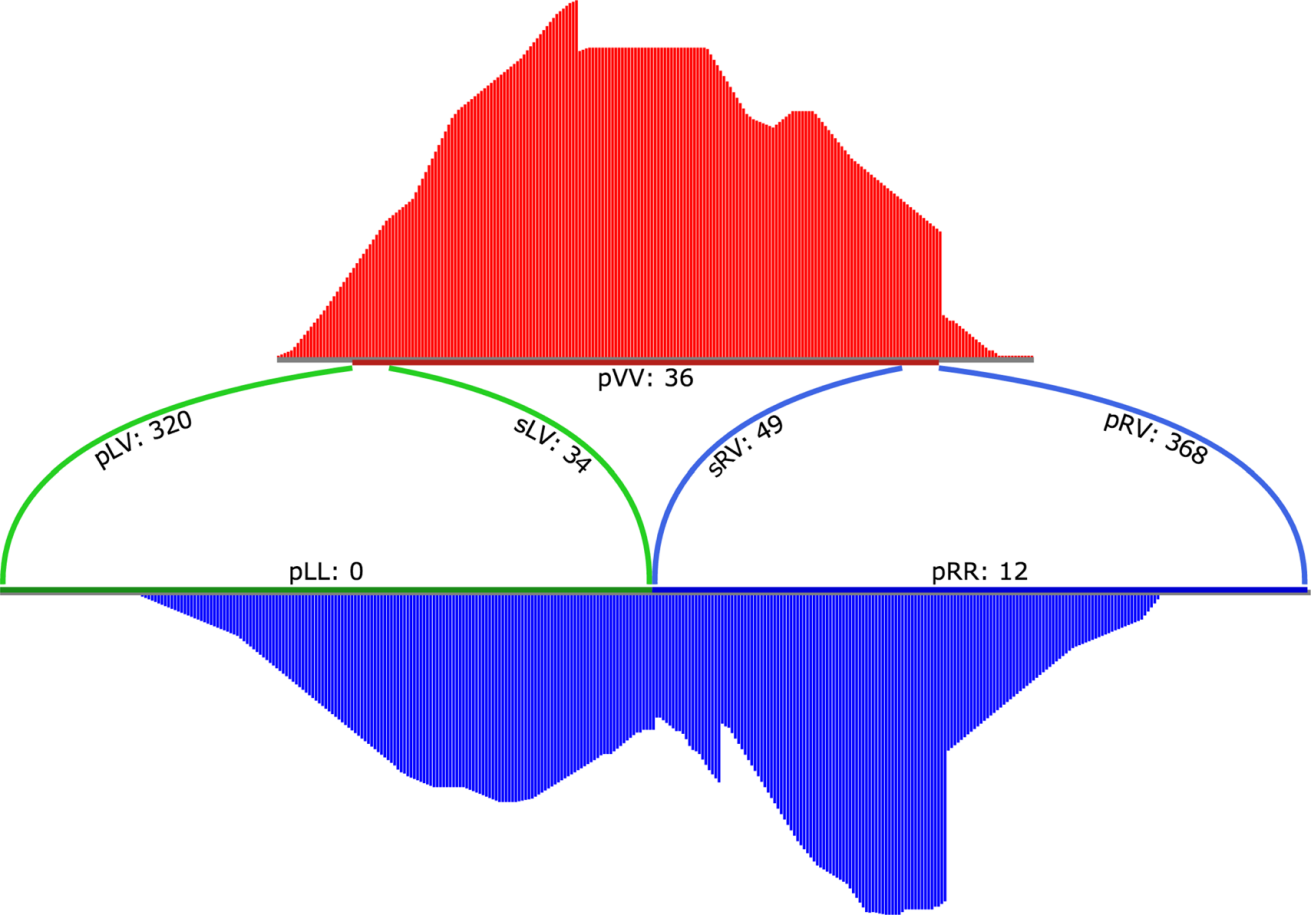
A demo plot of 1 viral integration cluster in its pre-insertion form.
  Here, D is the observation of the next-generation sequencing (NGS) reads on given position. P(Ti|D) is the likelihood component, which can be interpreted as the probability of observing D when the true genotype is T_i_. D_W_ is a realization (or observation) of the NGS reads in the Watson strand. D_C_ is a realization (or observation) of the NGS reads in the Crick strand. P(T_Wi_|D) is the likelihood component, which can be interpreted as the probability of observing D when the true genotype is T_Wi_. P(T_Ci_|D) is the likelihood component, which can be interpreted as the probability of observing D when the true genotype is T_Ci_. At each virus location, prior probability P(Ti) of each genotype Ti was set according to Table S5. The likelihood P(D|Ti) for the assumed genotype Ti was calculated from the observed allele types in the sequencing reads in formula 2. Thus, on the Watson strand, it is P(D_W_|T_i_), and on the Crick strand it is P(D_C_|T_i_). We defined the likelihood of observing allele d_k_ in a read for a possible haploid genotype T as P(d_k_|T), on the Watson strand it is P(d_Wk_|T), and on the Crick strand it is P(d_Ck_|T). So, for a set of n observed alleles at a locus, D = {d_1_, d_2_, …, d_n_} on each strand, these probabilities are computed as shown by formulas (3) and (4), where Q stands for the base quality from the fastaq file.
  (2) \begin{equation*}P({D_W}|{T_i}) = \prod\limits_{k = 1}^m {P({d_{Wk}}|T),P({D_C}|{T_i})} = \prod\limits_{k = 1}^n {P({d_{Ck}}|T)} .\end{equation*}
  (3) \begin{equation*}P({d_{Wk}}|T) = \left\{ {\begin{array}{@{}*{2}{c}@{}} \displaystyle {1 - {{10}^{ - \frac{Q}{{10}}}}}&{(T \in \{ A,C,G\} )}\nonumber\\ \displaystyle {\frac{{1 - {{10}^{ - \frac{Q}{{10}}}}}}{2}}&{(T \in \{ T\} )} \end{array},} \right.\end{equation*}
  (4) \begin{equation*}P({d_{Ck}}|T) = \left\{ {\begin{array}{@{}*{2}{c}@{}} \displaystyle {1 - {{10}^{ - \frac{Q}{{10}}}}}&{(T \in \{ C,G,T\} )}\nonumber\\ \displaystyle {\frac{{1 - {{10}^{ - \frac{Q}{{10}}}}}}{2}}&{(T \in \{ A\} )} \end{array}.} \right.\end{equation*}
  We used “Y” and “R” to represent C/T and G/A, respectively (IUPAC nucleotide code). If a region is covered by both the Watson strand and the Crick strand, we were able to deduce the original base from Y or R by calculation.
4. Detection of viral integrations: The assembled clipping regions above were mapped to the given virus reference sequence with a Smith-Waterman local alignment tool from the EMBOSS package [24], which supports IUPAC DNA codes Y and R. Virus fragment location is extracted from the alignment results.

## Discussion

In summary, we have implemented the first software tool to detect virus integration using BS data. Our software is based on bwa-meth, and by assembling and aligning soft-clip regions, it can find the virus breakpoints. However, accuracy of reads surrounding the breakpoints needs to be further improved. A virus usually integrates into regions that are homologous to both human and virus (micro-homologous) [25]. Therefore, we consider breakpoints predicted by our software tool correctly identified if they are within 10 bp of a real breakpoint (Fig. S2). With this definition, the accuracy of our predicted breakpoints can reach over 70%. Our results will be useful for analyzing BS data and related applications. Some of the results come with only a location on human genome, and the virus location missing. This may be due to the shortage of virus fragments. We simulated 3 kinds of reads, PE50, 90, and 150 with various lengths, and further simulated virus-inserted fragment with different lengths as well (Table S6); thus all cases described in Fig. 2 are mimicked here. All simulations sampled all possible reads, base by base with fixed insert sizes. As the result in Table S6 showed, the longer the reads, the more accurate a prediction can be achieved. In particular, for read lengths around 50 bp, BS-virus-finder is capable of finding the virus integration with an accuracy of more than 70%; for the read lengths between 90 bp and 150 bp, BS-virus-finder is capable of finding the virus integration with an accuracy of more than 90%. Apart from simulated data, we have performed WGS and WGBS sequencing of the PLC/PRF/5 hepatocellular carcinoma cell line (Table S4). As the results show, when the length of input is larger than 150 bp, the analysis result of WGBS is similar to the one of WGS. Additionally, BS-virus-finder is able to find breakpoints in 8 out of 9 regions identified by FISH [8]. Based on these experimental results, we believe that BS-virus-finder is a powerful software tool to analyze virus integration using BS data.

## Availability and requirements

Project Name: BS-virus-finder: virus integration calling using bisulfite-sequencing data

Project home page: https://github.com/BGI-SZ/BSVF [26]

Operating system: Linux

Programming language: Perl, Python, C

License: LGPL v3

Research Resource Identifier: BSVF, RRID:SCR_015727

## Availability of supporting data

Data used in this paper are simulated based on random insertion of the HBV sequence into the human chromosome 1 sequence. A Perl script named “simVirusInserts.pl” is included, and our simulation schema is coded within. We have run the simulation several times, and the result shows no significant difference. The PLC/PRF/5 hepatocellular carcinoma cell lines were from American Type Culture Collection (ATCC, Manassas, VA, USA) and sequenced by HiSeq X Ten System from Novogene company. WGS and WGBA data have been submitted to NCBI SRA project PRJNA400455. Supporting data, an archival copy of the code, and the Perl script “simVirusInserts.pl” are also available via the *GigaScience* repository, *Giga*DB [27].

## Additional files

Additional Table S1: Alignment accuracy rate around the breakpoint region using PE50 data.

Additional Table S2: Alignment accuracy rate around the breakpoint region using PE90 data.

Additional Table S3: Alignment accuracy rate around the breakpoint region using PE150 data.

Additional Table S4: Mapping statistics of cell line sequencing data.

Additional Table S5: The prior probability of the Bayesian model used in the restoring process for bisulfite sequencing of integrated virus.

Additional Table S6: The performance of BS-virus-finder *in silico* with different read lengths and insert sizes.

Additional Figure S1: The performance of BS-virus-finder in various lengths of virus integration using PE50.

Additional Figure S2: The performance of BS-virus-finder in various lengths of virus integration using PE90.

Additional Figure S3: The performance of BS-virus-finder in various lengths of virus integration using PE150.

Additional Figure S4: The diagram of STR for the PLC/PRF/5 cell line.

## Abbreviations

bp: base pair; BS: bisulfite sequencing; DMR: different methylation region; HBV: hepatitis B virus; IUPAC: International Union of Pure and Applied Chemistry; NGS: next-generation sequencing; PCR: polymerase chain reaction; PE: paired-end; SNP: single nucleotide polymorphism; WGBS: Whole-genome-based bisulfite sequencing.

## Competing interests

The authors declare that they have no competing interests.

## Funding

This work was funded by the National Natural Science Foundation of China (81602477) and Shenzhen Municipal Government of China (ZDSYS201507301424148).

## Authors contributions

C.P., L.B., and H.Y. conceptualized the project. S.G., X.H., S.L., and J.W. designed BSVF and developed its accompanying utilities. S.G., X.H., C.G., X.Z., M.W., and S.Z. developed the protocol. F.X., D.F., H.C., and J.B. conducted experiments. S.G., X.H., B.L., and S.W. undertook the analysis. K.X., L.M., S.G., X.H., L.B., and C.P. wrote and approved the final version of the manuscript. All authors read and approved the final manuscript.

## Supplementary Material

GIGA-D-17-00032_Original_Submission.pdf

GIGA-D-17-00032_Revision_1.pdf

GIGA-D-17-00032_Revision_2.pdf

GIGA-D-17-00032_Revision_3.pdf

Response_to_Reviewer_Comments_Original_Submission.pdf

Response_to_Reviewer_Comments_Revision_1.pdf

Response_to_Reviewer_Comments_Revision_2.pdf

Reviewer_1_Report_(Original_Submission) -- Lada Koneva 07 Mar 2017 Reviewed

Reviewer_1_Report_(Revision_1) -- Lada Koneva 22 Sep 2017 Reviewed

Reviewer_1_Revision_1_(Attachment).pdf

Reviewer_2_Report_(Original_Submission) -- Thomas Mikeska 07 Mar 2017 Reviewed

Reviewer_2_Report_(Revision_1) -- Thomas Mikeska 12 Sep 2017 Reviewed

Supplemental material
