# Supplementary material for: BS-virus-finder: virus integration calling using bisulfite sequencing data
Source: Gigascience. 2017 Dec 18;7(1):1–7. doi: 10.1093/gigascience/gix123 (PMC5788064; doi:10.1093/gigascience/gix123)
Supplement: GIGA-D-17-00032_Original_Submission.pdf [file gix123_giga-d-17-00032_original_submission.pdf]

GigaScience

BS-virus-finder: virus integration calling using bisulfite-sequencing data

--Manuscript Draft--

**Manuscript Number:**	GIGA-D-17-00032
**Full Title:**	BS-virus-finder: virus integration calling using bisulfite-sequencing data
**Article Type:**	Data Note
**Funding Information:**	Young Scientists Fund of the National Natural Science Foundation of China (009142) — Dr. Shengjie Gao
**Abstract:**	Background: DNA methylation plays a key role in regulating gene expression and carcinogenesis. Extant methylation bisulfite sequencing (BS) researches mainly focus on calling SNP, DMR, and ASM, instead of virus integration positions.<br>Findings: We developed a new and easy-to-use software, named as BS-virus-finder (https://github.com/BioInfoTools/BSVF), to detect viral integration breakpoints in whole human genomes.<br>Conclusions: BS-virus-finder demonstrates moderate sensitivity and specificity, and is useful to be applied in epigenetic researches and to reveal the relationship between viral integration and DNA methylation. BS-virus-finder is the first software to detect virus by using bisulfite sequencing data.
**Corresponding Author:**	Christian Pedersen<br><br>DENMARK
**Corresponding Author Secondary Information:**	
**Corresponding Author's Institution:**	
**Corresponding Author's Secondary Institution:**	
**First Author:**	Shengjie Gao, Ph.D
**First Author Secondary Information:**	
**Order of Authors:**	Shengjie Gao, Ph.D
	Xuesong Hu
	Changduo Gao
	Kai Xiong
	Shancen Zhao
	Mengyao Wang
	Xiaohui Zhao
	Jie Bai
	Bo Li
	Song Wu
	Shengbin Li
	Huanming Yang
	Lars Bolund
	Christian Pedersen
**Order of Authors Secondary Information:**	
**Opposed Reviewers:**	

Powered by Editorial Manager® and ProduXion Manager® from Aries Systems Corporation

Additional Information:	
**Question**	**Response**
Are you submitting this manuscript to a special series or article collection?	No
**Experimental design and statistics**<br><br>Full details of the experimental design and statistical methods used should be given in the Methods section, as detailed in our Minimum Standards Reporting Checklist. Information essential to interpreting the data presented should be made available in the figure legends.<br><br>Have you included all the information requested in your manuscript?	Yes
**Resources**<br><br>A description of all resources used, including antibodies, cell lines, animals and software tools, with enough information to allow them to be uniquely identified, should be included in the Methods section. Authors are strongly encouraged to cite Research Resource Identifiers (RRIDs) for antibodies, model organisms and tools, where possible.<br><br>Have you included the information requested as detailed in our Minimum Standards Reporting Checklist?	Yes
**Availability of data and materials**<br><br>All datasets and code on which the conclusions of the paper rely must be either included in your submission or deposited in publicly available repositories (where available and ethically appropriate), referencing such data using a unique identifier in the references and in the “Availability of Data and Materials” section of your manuscript.<br><br>Have you have met the above requirement as detailed in our Minimum Standards Reporting Checklist?	Yes

Powered by Editorial Manager® and ProduXion Manager® from Aries Systems Corporation

Manuscript

Click here to download Manuscript BS-virus-finder-manuscript.docx

BS-virus-finder: virus integration calling using bisulfite-sequencing data

Shengjie Gao[1,2,7,8,9*], Xuesong Hu[2*], Changduo Gao[3*], Kai Xiong[4*], Shancen Zhao[5,7], Mengyao Wang[5], Xiaohui Zhao[6], Jie Bai[5], Bo Li[2], Song Wu[9], Shengbin Li[2], Huangming Yang[5, 7#], Lars Bolund[8#], Christian N. S. Pedersen[1#]

[1] Bioinformatics Research Centre, Aarhus University, Aarhus, Denmark

[2] BGI-Forensic, Shenzhen 518083, China

[3] College of Computer Science & Technology, Qingdao University, Qingdao 266071, China

[4] Department of Veterinary Clinical and Animal Sciences, University of Copenhagen, Copenhagen, Denmark

[5] BGI-Shenzhen, Shenzhen 518083, China

[6] College Of Mathematics & Statistics, Changsha University of Science & Technology, Changsha 410114, China

[7] James D. Watson Institute of Genome Sciences, Hangzhou 310058, China

[8] Department of Biomedicine, Aarhus University, Aarhus, Denmark

[9] The Affiliated Luohu Hospital of Shenzhen University, Shenzhen University, Shenzhen 518000, China.

[*] These authors contributed equally to this work

[#] These authors equally directed the work

Abstract:

Background: DNA methylation plays a key role in regulating gene expression and carcinogenesis. Extant methylation bisulfite sequencing (BS) researches mainly focus on calling SNP, DMR, and ASM, instead of virus integration positions.

Findings: We developed a new and easy-to-use software, named as BS-virus-finder (https://github.com/BioInfoTools/BSVF), to detect viral integration breakpoints in whole human genomes.

Conclusions: BS-virus-finder demonstrates moderate sensitivity and specificity, and is useful to be applied in epigenetic researches and to reveal the relationship between viral integration and DNA methylation. BS-virus-finder is the first software to detect virus by using bisulfite sequencing data.

Keyword: Virus integration, Bisulfite sequencing, Carcinogenesis

Findings

Introduction

DNA methylation plays crucial roles in many areas including development [3, 4] and X chromosome inactivation [5] by regulating genetic imprinting and epigenetic modification without altering DNA sequences. Previous researches showed strong association of DNA methylation with cancer. The methylation status altering related carcinogenesis [6], cancer recurrence [7] and metastasis [8] were already revealed by emerging bisulfite sequencing technology (BS). BS technology can investigate DNA methylation changes with the single-base accuracy. Treatment of DNA with bisulfite converts cytosine residues to uracil, but leaves 5-methylcytosine residues unmodified [9]. Thus, bisulfite treatment introduces specific changes in the DNA sequence that depend on the methylation status of individual cytosine residues, yielding single-nucleotide resolution information about the methylation status of a segment of DNA. Various analyses can be performed on the altered sequences to retrieve this information. BS technology can reveal differences between SNP (cytosines and thymidine) and sequence change resulting from bisulfite conversion. Whole-genome based bisulfite sequencing (Bis-seq) has been developed to detect DNA methylation. A recent clinical study showed that DNA methylation is associated with viral integration [10]. Whole-genome BS (WGBS) data can be analyzed to investigate the sequence mapping and alignment via BSMAP [11], Bismark [12] and BWA-meth [13], to detect DMR via software QDMR [14], DMAP [15] and SMAP [11], to identify SNP via software BS-SNPer [2] and Bis-SNP [16], and finding ASM via SMAP [1],

Methy-Pipe [17]. However, none of them can be used for virus integration loci calling, and no software is currently available to detect virus integration loci by analyzing BS data. Therefore, we developed the software to detect the virus integration loci by genome-wide BS analysis.

Data description in silico

PE reads (90bp) that include 700 breakpoints in chromosome 18 (chr.18) of GRCh38 were simulated in our study. Input fragments of 50 to 400 bp were randomly selected from chromosome 18 in the GRCh37 assembly (hg19) of the human genome. The length of viral integration was between 45bp to 180 bp. After the alignment, the mapping accuracy of each of the 17 different types of reads mapping was calculated. Mapping accuracy varied among the 17 types of read mappings in our simulation (Figure S1, S2, S3). In summary, the accuracies of several kinds of the reads mappings were low (Table S1, S2, S3), which may raise false-negative rate. Generally, however, bwa-meth [13] performed very well. It indicated virus breakpoints might be hardly found by our BS virus finder.

Bisulfite sequencing is a marvelous and sophisticated technique to study DNA cytosine methylation. Bisulfite treatment followed by PCR amplification specifically converts methylated cytosine to thymine. By cooperating with next generation sequencing technology, it is able to detect the methylation status of every cytosine in the whole genome. Moreover, longer as the read is, higher accuracy can be achieved.

Method for calling virus integration

Four steps were implemented to obtain virus integration:

1. Alignment

The first step is alignment. We used Bwa-meth to align junction reads and mark the shorter junction parts as soft-clip, which enables us to find breakpoints directly from the alignment.

2. Clustering

After the alignment, the result was filtered based on sequencing quality, mapping quality and mismatch rates. Then, all reads surrounding or containing breakpoints were identified to form clusters. As the figure shows (Figure 1), there are 17 kinds of mapping reads with the information of viral integration. Each cluster contains one or more of such reads. Clusters are extended until no more overlapped paired end (PE) reads found.

3. Assembling

Based on the results of clustering, we identified the most likely candidate breakpoints to predict the most possible virus sequence candidates. Within these candidates, our restore algorithm was used to calculate the most possible base in each region, and then to find the integration region of virus (Figure 2). Furthermore, we can calculate maximum a posteriori probability estimate for A, C, G, T as:

$$\begin{aligned} P(T_i \mid D) &= \frac{P(T_{Wi})P(D \mid T_{Wi})}{\sum_{x=1}^{S} P(T_{Wx})P(D \mid T_{Wx})} \times \frac{P(T_{Ci})P(D \mid T_{Ci})}{\sum_{x=1}^{S} P(T_{Cx})P(D \mid T_{Cx})} \\ &= C_0 \times P(D \mid T_{Wi}) \times P(D \mid T_{Ci}) \\ C_0 &= \frac{P(T_{Wi})}{\sum_{x=1}^{S} P(T_{Wx})P(D \mid T_{Wx})} \times \frac{P(T_{Ci})}{\sum_{x=1}^{S} P(T_{Cx})P(D \mid T_{Cx})} \end{aligned}$$

.

D be a realization (or observation) of the NGS reads. P(Ti|D) is the likelihood component, which can be interpreted as the probability of observing D when the true genotype is T_i. D_W be a

realization (or observation) of the NGS reads in Watson strand. D_C be a realization (or observation) of the NGS reads in Crick strand. $P(T_{Wi}|D)$ is the likelihood component, which can be interpreted as the probability of observing D when the true genotype is T_{Wi}. $P(T_{Ci}|D)$ is the likelihood component, which can be interpreted as the probability of observing D when the true genotype is T_{Ci}. At each genomic location, prior probability $P(T_i)$ of each genotype T_i was set according to the reference genotype. The likelihood $P(D|T_i)$ for the assumed genotype T_i was calculated from the observed allele types in the sequencing reads. Thus, on Watson strand it is $P(D_W|T_i)$, on Crick strand it is $P(D_C|T_i)$. We defined the likelihood of observing allele d_k in a read for a possible haploid genotype T as $P(d_k|T)$, and on Watson strand it is $P(d_{Wk}|T)$, on Crick strand it is $P(d_{Ck}|T)$. So, for a set of n total observed alleles at a locus, $D = \{d_1, d_2, \ldots, d_n\}$ on each strand.

$$P(D_W \mid T_i) = \prod_{k=1}^{m} P(d_{Wk} \mid T), P(D_C \mid T_i) = \prod_{k=1}^{n} P(d_{Ck} \mid T).$$

$$P(d_{Wk} \mid T) = \begin{cases} 1 - 10^{-\frac{Q}{10}} & (T \in \{A, C, G\}) \\ \dfrac{1 - 10^{-\frac{Q}{10}}}{2} & (T \in \{T\}) \end{cases},$$

$$P(d_{Ck} \mid T) = \begin{cases} 1 - 10^{-\frac{Q}{10}} & (T \in \{C, G, T\}) \\ \dfrac{1 - 10^{-\frac{Q}{10}}}{2} & (T \in \{A\}) \end{cases}.$$

For the bases without methylation, and all G changed to A on Crick strand. Thus we used “Y” and “R” to represent C/T and G/A respectively (IUPAC nucleotide code). If a region is covered by both Watson strand and Crick strand, we were able to reveal the original base from Y or R by calculation. The unmapped regions above hereby mapped to the given virus reference sequence with the Smith-Waterman local alignment tool [18], which support IUPAC DNA codes. Virus fragment location is extracted from the alignment result. As shown in Figure 3.

4. Detection of viral integrations

The unmapped regions were thereby mapped to the given virus reference sequence with the Smith-Waterman local alignment tool [18], which support IUPAC DNA codes. Virus fragment location is extracted from the alignment results.

Discussion

In summary, we implemented the first software to detect virus integration using BS data. Our software is based on Bwa-meth. By identifying soft-clip, it can easily find the virus breakpoints. However, accuracy of reads surrounding the breakpoints needs to be further improved. Virus usually integrates into regions that homologous to both human and virus (micro-homologous). Therefore, the breakpoints predicted by our software within the nearest 10 bp around the real breakpoint were considered as the perfect results (Figure S2). The accuracy of predicted breakpoints can reach over 70%. Our results will be useful for analyzing BS data and relative applications. Some of the results come with only location on human genome, and the virus location is missing. This may due to the shortage of virus fragments. We stimulated three kinds of reads, PE50, 90,150 with various length, and further stimulated virus-inserted fragment with different length as well (Table 1), thus all cases described in Figure 1 are mimic here. As the result in Table 1 showed, the longer the reads, the more accurate the prediction can be achieved. Particularly, the result in Table1 demonstrated that Bs-virus-finder is capable to find more than

80% of virus integration with the accuracy more than 90%. Taking together, we hereby provided a powerful tool to analyze virus-integration using BS data.

Availability and requirements

Project Name: BS-virus-finder: virus integration calling using bisulfite-sequencing data

Project home page: https://github.com/BioInfoTools/BSVF

Operating system: Linux

Programming language: Perl and Python

License: GPL v3

Availability of supporting data

Data used in this paper is simulated based on random insertion of HBV sequence to human chromosome 1 sequence. A Perl script named "simVirusInserts.pl" is included, and our simulation schema is coded within. We have run the simulation several times and the result shows no significant difference.

Competing interests

The authors declare that they have no competing interests.

Acknowledgements

We appreciate the supporting of Xiaolin Liang and Hengtong Li in College of Mathematics & Statistics, Changsha University of Science & Technology, for their contributing advice to our

research. This work was supported financially by the Young Scientists Fund of the National Natural Science Foundation of China (grant no. 009142).

References:

1. Gao S, Zou D, Mao L, Zhou Q, Jia W, Huang Y, Zhao S, Chen G, Wu S, Li D *et al*: **SMAP: a streamlined methylation analysis pipeline for bisulfite sequencing**. *GigaScience* 2015, **4**:29.
2. Gao S, Zou D, Mao L, Liu H, Song P, Chen Y, Zhao S, Gao C, Li X, Gao Z *et al*: **BS-SNPer: SNP calling in bisulfite-seq data**. *Bioinformatics* 2015, **31**(24):4006-4008.
3. Wang Y, Shang Y: **Epigenetic control of epithelial-to-mesenchymal transition and cancer metastasis**. *Experimental cell research* 2013, **319**(2):160-169.
4. O'Doherty AM, Magee DA, O'Shea LC, Forde N, Beltman ME, Mamo S, Fair T: **DNA methylation dynamics at imprinted genes during bovine pre-implantation embryo development**. *BMC developmental biology* 2015, **15**:13.
5. Cotton AM, Price EM, Jones MJ, Balaton BP, Kobor MS, Brown CJ: **Landscape of DNA methylation on the X chromosome reflects CpG density, functional chromatin state and X-chromosome inactivation**. *Human molecular genetics* 2015, **24**(6):1528-1539.
6. Kamdar SN, Ho LT, Kron KJ, Isserlin R, van der Kwast T, Zlotta AR, Fleshner NE, Bader G, Bapat B: **Dynamic interplay between locus-specific DNA methylation and hydroxymethylation regulates distinct biological pathways in prostate carcinogenesis**. *Clinical epigenetics* 2016, **8**:32.
7. Haldrup C, Mundbjerg K, Vestergaard EM, Lamy P, Wild P, Schulz WA, Arsov C, Visakorpi T, Borre M, Hoyer S *et al*: **DNA methylation signatures for prediction of biochemical recurrence after radical prostatectomy of clinically localized prostate cancer**. *Journal of clinical oncology : official journal of the American Society of Clinical Oncology* 2013, **31**(26):3250-3258.
8. Kim JH, Dhanasekaran SM, Prensner JR, Cao X, Robinson D, Kalyana-Sundaram S, Huang C, Shankar S, Jing X, Iyer M *et al*: **Deep sequencing reveals distinct patterns of DNA methylation in prostate cancer**. *Genome research* 2011, **21**(7):1028-1041.
9. Darst RP, Pardo CE, Ai L, Brown KD, Kladde MP: **Bisulfite sequencing of DNA**. *Current protocols in molecular biology / edited by Frederick M Ausubel [et al]* 2010, **Chapter 7**:Unit 7 9 1-17.
10. Lillsunde Larsson G, Helenius G, Sorbe B, Karlsson MG: **Viral load, integration and methylation of E2BS3 and 4 in human papilloma virus (HPV) 16-positive vaginal and vulvar carcinomas.** *PloS one* 2014, **9**(11):e112839.

11. Xi Y, Li W: **BSMAP: whole genome bisulfite sequence MAPping program**. *BMC bioinformatics* 2009, **10**:232.

12. Krueger F, Andrews SR: **Bismark: a flexible aligner and methylation caller for Bisulfite-Seq applications**. *Bioinformatics* 2011, **27**(11):1571-1572.

13. Pedersen BS EK, De S, Yang IV, Schwartz DA: **Fast and accurate alignment of long bisulfite-seq reads**. *eprint arXiv* 2014, **14011129**

14. Zhang Y, Liu H, Lv J, Xiao X, Zhu J, Liu X, Su J, Li X, Wu Q, Wang F *et al*: **QDMR: a quantitative method for identification of differentially methylated regions by entropy**. *Nucleic acids research* 2011, **39**(9):e58.

15. Stockwell PA, Chatterjee A, Rodger EJ, Morison IM: **DMAP: differential methylation analysis package for RRBS and WGBS data**. *Bioinformatics* 2014.

16. Liu Y, Siegmund KD, Laird PW, Berman BP: **Bis-SNP: Combined DNA methylation and SNP calling for Bisulfite-seq data**. *Genome biology* 2012, **13**(7):R61.

17. Jiang P, Sun K, Lun FM, Guo AM, Wang H, Chan KC, Chiu RW, Lo YM, Sun H: **Methy-Pipe: an integrated bioinformatics pipeline for whole genome bisulfite sequencing data analysis**. *PloS one* 2014, **9**(6):e100360.

18. Rice P, Longden I, Bleasby A: **EMBOSS: the European Molecular Biology Open Software Suite**. *Trends in genetics : TIG* 2000, **16**(6):276-277.

Table 1. The performance of BS-virus-finder.

Read Length	PE Insert Length	Virus Length	True Positive Cnt	True Positive %	HumOnly Cnt	HumOnly %	VirNA Cnt	VirNA %	HumCalled %	False Positive Cnt	False Positive %
**50**	60	25	454	64.86%	22	3.14%	119	17.00%	85.00%	1	0.14%
	80	25	438	62.57%	30	4.29%	174	24.86%	91.71%	7	1.00%
	80	67	640	91.43%	2	0.29%	0	0.00%	91.71%	4	0.57%
	120	5	51	7.29%	479	68.43%	52	7.43%	83.14%	1	0.14%
	120	10	76	10.86%	428	61.14%	160	22.86%	94.86%	9	1.29%
	120	20	512	73.14%	29	4.14%	131	18.71%	96.00%	1	0.14%
	120	25	450	64.29%	28	4.00%	191	27.29%	95.57%	3	0.43%
	120	67	581	83.00%	13	1.86%	74	10.57%	95.43%	3	0.43%
	120	100	673	96.14%	1	0.14%	0	0.00%	96.29%	11	1.57%
	250	67	262	37.43%	266	38.00%	20	2.86%	78.29%	0	0.00%
**90**	100	45	569	81.29%	0	0.00%	109	15.57%	96.86%	60	8.57%
	150	45	570	81.43%	2	0.29%	115	16.43%	98.14%	38	5.43%
	150	120	585	83.57%	11	1.57%	94	13.43%	98.57%	72	10.29%
	200	5	45	6.43%	539	77.00%	66	9.43%	92.86%	0	0.00%
	200	10	213	30.43%	295	42.14%	151	21.57%	94.14%	1	0.14%
	200	20	498	71.14%	37	5.29%	123	17.57%	94.00%	3	0.43%
	200	45	570	81.43%	1	0.14%	116	16.57%	98.14%	24	3.43%
	200	120	692	98.86%	0	0.00%	0	0.00%	98.86%	78	11.14%
	200	180	616	88.00%	8	1.14%	65	9.29%	98.43%	71	10.14%
	420	120	689	98.43%	0	0.00%	0	0.00%	98.43%	30	4.29%
**150**	150	75	477	68.14%	6	0.86%	209	29.86%	98.86%	97	13.86%
	220	75	576	82.29%	1	0.14%	114	16.29%	98.71%	57	8.14%
	220	200	691	98.71%	0	0.00%	0	0.00%	98.71%	68	9.71%
	350	5	50	7.14%	554	79.14%	67	9.57%	95.86%	0	0.00%
	350	10	69	9.86%	447	63.86%	158	22.57%	96.29%	0	0.00%
	350	20	513	73.29%	37	5.29%	126	18.00%	96.57%	2	0.29%
	350	75	474	67.71%	6	0.86%	212	30.29%	98.86%	42	6.00%

350	200	691	98.71%	0	0.00%	0	0.00%	98.71%	43	6.14%
350	300	678	96.86%	10	1.43%	2	0.29%	98.57%	62	8.86%
530	200	691	98.71%	0	0.00%	0	0.00%	98.71%	35	5.00%

* We simulated 700 virus insertion events in each row.
Correct: Distance between simulated and found point is within 10 bp range.
True Positive: Both human split site and virus split site are correct. False Positive: Human split site is wrong.
HumOnly: Human split site is right but virus split site is wrong.
VirNA: Human split site is right but virus split site is not found.
HumCalled: Sum of left (all listed except for FP), which is the called rate on human genome.

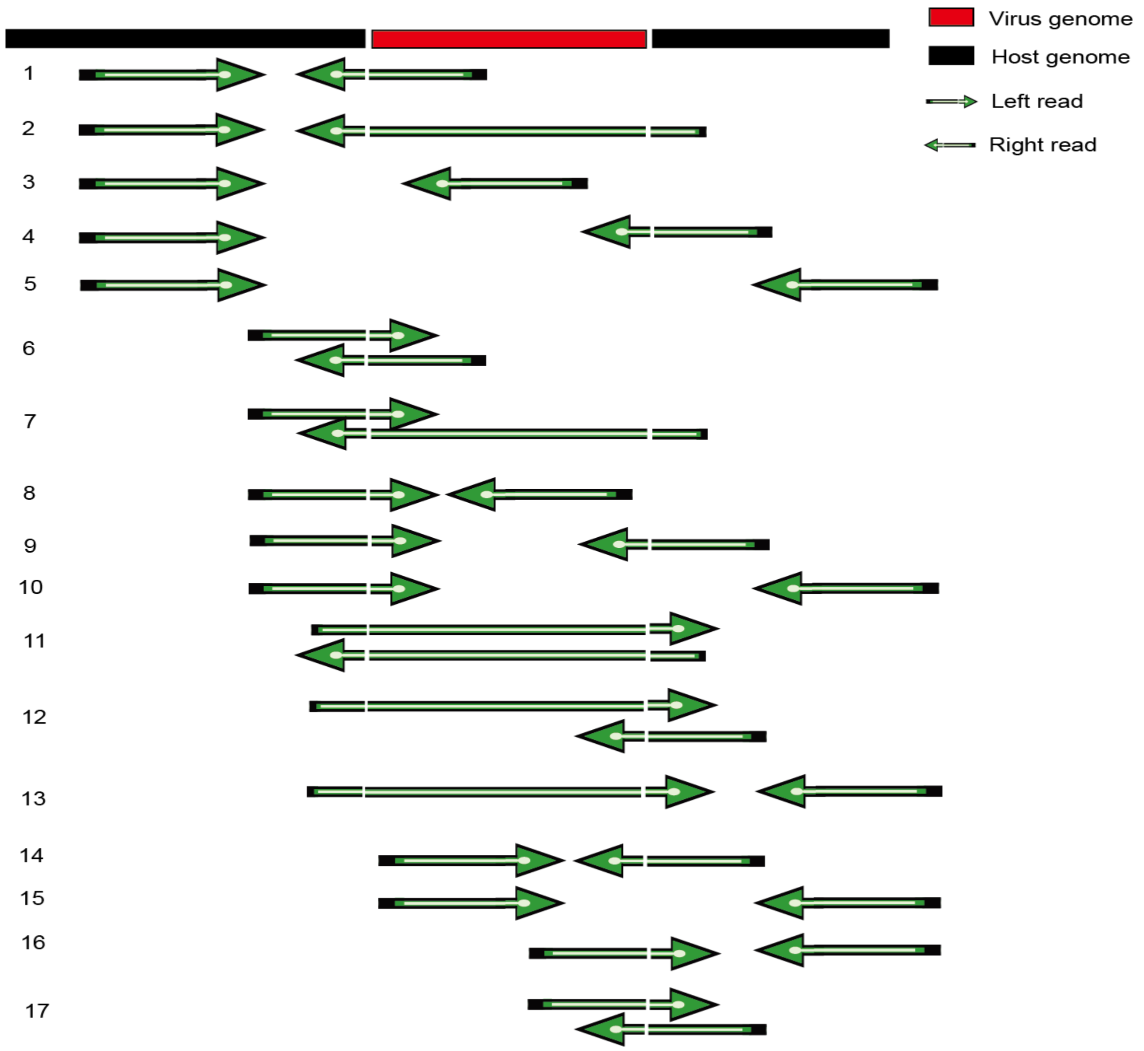

Figure 1. Principal types of mapping reads around the viral integration site.

Red bar, the virus sequence inserted in host genome; Green arrow, mapping reads with different directions; Breakpoints indicate logical division between host genome and virus, which are physically linked.

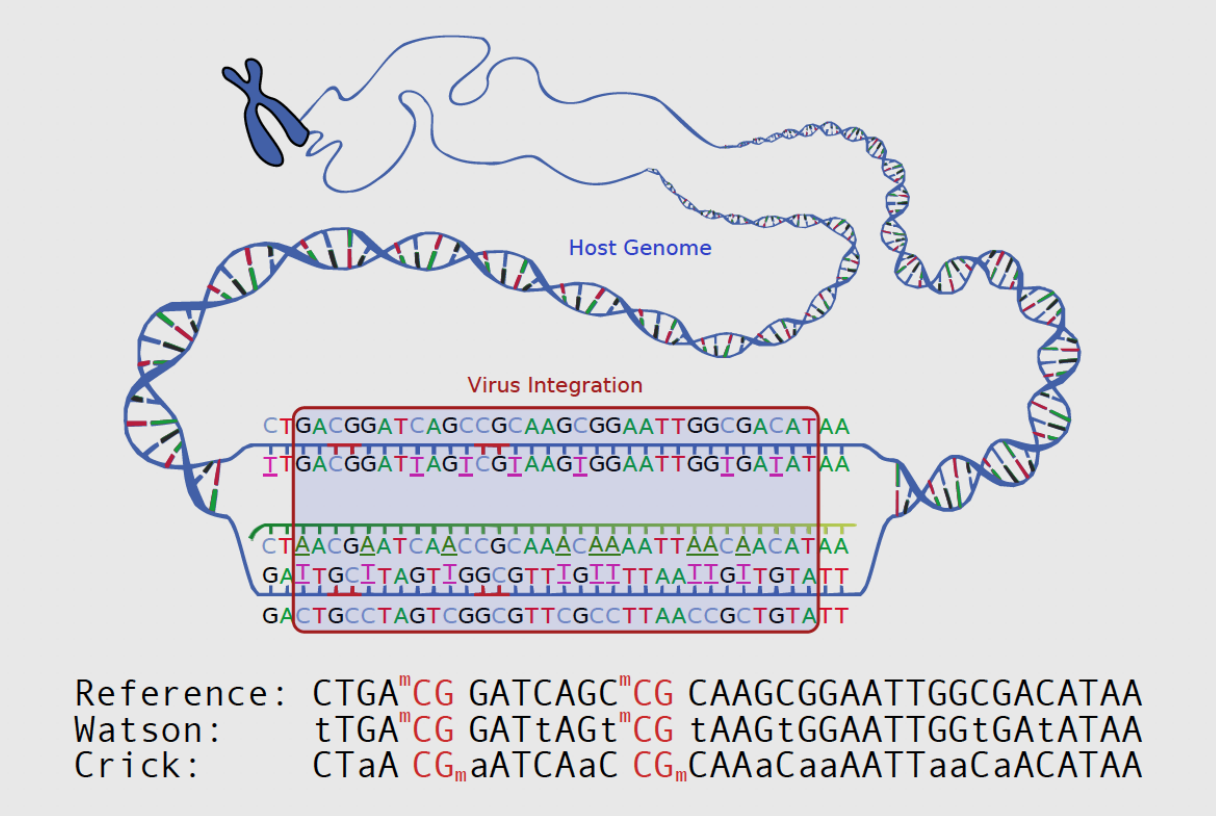

Figure 2. The illustration of restore algorithm restoring the bisulfite-altered sequence to the original.

This is a typical alignment of BS reads to the virus integration sites. Methylation sites were showed as read bases. Bisulfite treated base may alter the original base from G to A, or C to T. Our restore algorithm was used to reveal the original sequence after BS data clustering. m indicated methylation-modified base. Low-case letter indicates the unmatched base to the reference.

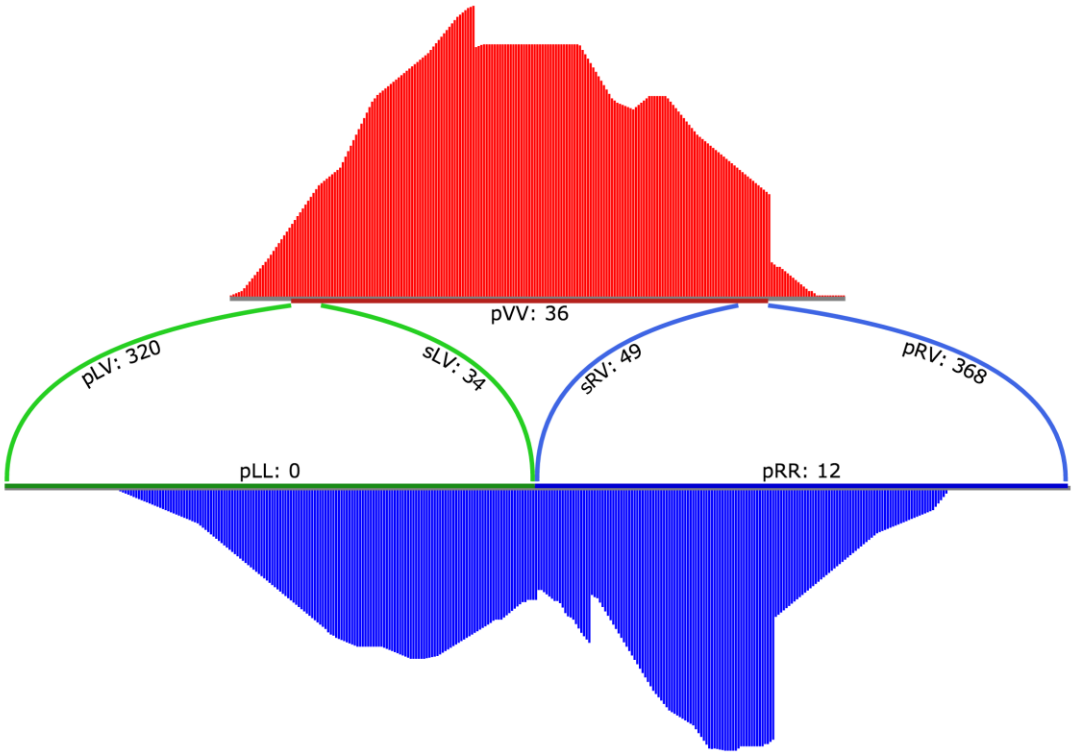

Figure 3. A simulated model of viral integration cluster around breakpoints.

Horizon Lines： Red, Inserted virus fragments (V); Green zone, 5’ upstream of insertion (Left); Blue: 3’ downstream of insertion (Right); Gray, DNA strands;

Bars show the coverage depth on virus (red) and human genome (blue);

Curves show count of pair-end relationship of reads among Left, Right and Virus part: pLL, L to L pair-end reads; pLV, L to V pair-end reads; sLV, single reads mapped on both L and V.

Click here to access/download
Supplementary Material
Supplementary-bsfinder-0210.docx
