# Supplementary material for: BS-virus-finder: virus integration calling using bisulfite sequencing data
Source: Gigascience. 2017 Dec 18;7(1):1–7. doi: 10.1093/gigascience/gix123 (PMC5788064; doi:10.1093/gigascience/gix123)
Supplement: GIGA-D-17-00032_Revision_1.pdf [file gix123_giga-d-17-00032_revision_1.pdf]

GigaScience

BS-virus-finder: virus integration calling using bisulfite-sequencing data

--Manuscript Draft--

**Manuscript Number:**	GIGA-D-17-00032R1
**Full Title:**	BS-virus-finder: virus integration calling using bisulfite-sequencing data
**Article Type:**	Technical Note
**Funding Information:**	Young Scientists Fund of the National Natural Science Foundation of China (81602477) — Dr. Shengjie Gao<br>Shenzhen Municipal Government of China (ZDSYS201507301424148) — Prof. Shengbin Li
**Abstract:**	Background: DNA methylation plays a key role in regulating gene expression and carcinogenesis. Extant methylation bisulfite sequencing (BS) researches mainly focus on calling SNP, DMR, and ASM, instead of virus integration positions.<br>Findings: We developed a new and easy-to-use software, named as BS-virus-finder (https://github.com/BioInfoTools/BSVF), to detect viral integration breakpoints in whole human genomes.<br>Conclusions: BS-virus-finder demonstrates moderate sensitivity and specificity, and is useful to be applied in epigenetic researches and to reveal the relationship between viral integration and DNA methylation. BS-virus-finder is the first software to detect virus by using bisulfite sequencing data.
**Corresponding Author:**	Christian Pedersen<br><br>DENMARK
**Corresponding Author Secondary Information:**	
**Corresponding Author's Institution:**	
**Corresponding Author's Secondary Institution:**	
**First Author:**	Shengjie Gao, Ph.D
**First Author Secondary Information:**	
**Order of Authors:**	Shengjie Gao, Ph.D<br>Xuesong Hu<br>Changduo Gao<br>Kai Xiong<br>Fengping Xu<br>Xiao Zhao<br>Haixiao Chen<br>Shancen Zhao<br>Mengyao Wang<br>Dongke Fu<br>Xiaohui Zhao<br>Jie Bai<br>Bo Li<br>Song Wu<br>Shengbin Li

Powered by Editorial Manager® and ProduXion Manager® from Aries Systems Corporation

	Huanming Yang
	Lars Bolund
	Christian Pedersen
**Order of Authors Secondary Information:**	
**Response to Reviewers:**	Reviewer #1: Major Concerns<br>1.Why did the authors choose whole-genome bisulfite sequencing (WGBS) data for identification of the virus integration loci? There are many established tools or pipelines for detection of viral integration loci based on whole-genome sequencing (WGS) and/or RNA-seq. What is the rationale for developing a method using WGBS instead of improving upon the methods for WGS or RNA-seq for virus integration detection? Authors provide a reference, saying, "A recent clinical study showed that DNA methylation is associated with viral integration", but the work of Larsson GL et al, 2014 was not performed on whole-genome methylation data and hardly could be used as a rationale for using WGBS for the virus detection.<br><br>Thank you for your comments. There is no existing method for analysis of virus integration by using WGBS data unless additional relative WGS and RNA-seq data is available, thus requiring more human and financial resources. Regarding this, our software tool solved this problem by finding virus integration directly from WGBS data, therefore enabling efficiently and deeply mining data. We cite the work of Larsson GL to show that there is a relationship between virus integration and DNA methylation, and not as a rationale for using WGBS.<br><br>2.Authors listed several tools for the alignment of WGBS data on page 5 lines 18-20. Why did the authors use BWA-meth instead of another published tool? BWA-meth is not published in a peer-reviewed journal, whereas other aligners such as Bismark are, so authors should provide a rationale for choosing this aligner.<br><br>Soft clipping information was required when we initiate our search for the virus-integrated sites. However, there is no such function that can be used to provide this information by using the previous software (neither bsmap nor bismark) for sequence comparison. Therefore, we chose bwameth and bsseeker2 for the sequence comparison and we found BWA-meth showed the best. We finally applied BWA-meth as the software for comparision.<br><br>3. Simulation should be described/performed better. For example, what bisulfite conversion rate for cytosines in CG-context was used in this simulation? There are tools for bisulfite read simulation, for example, SHERMAN which allows users to simulate bisulfite reads with varying bisulfite conversion rate.<br><br>We applied SHERMAN to randomly model 100 break points with 20% conversation rate. Out of 94 break points, 89 are correct, 5 are false positive., This result is similar with our preliminary modeling result.<br><br>4. It is not clear from the manuscript whether authors tried to find real data for testing BS-virus-finder. The authors should include results using real WGBS data in addition to simulated data. If there is no such data, then there is no reason to develop a method for it.<br><br>We performed WGS and WGBS for PLC/PRF/5 cell line and anaylizer the data. The result is showed in Table 1.<br><br>5. Performance of BS-virus-finder should be compared with performance of the existing tools/pipelines for detection viral integration on WGS and RNA-seq (for example VirusSeq). Authors could remove BS-conversion from their simulated data and use them for running VirusSeq or other established pipeline for virus detection. But using real data would be better.<br><br>We performed WGS and WGBS for PLC/PRF/5 cell line and anaylizer the data. The result is showed in Table 1.<br><br>6. The section "Method for calling virus integration" is written from the point of view that

authors know which reads contain junctions. This is could be true for simulated data but not for real data. It raises many questions through the Methods section - how will it work on real data? Probably authors should re-write text in the context of working with real (or proper simulated) reads, when users do not know which reads contain junctions.

We performed WGC and WGBS for PLC/PRF/5 cell line and detected by BSVF, respectively. The result is showed in Table 1.

7. In the sentence "We used Bwa-meth to align junction reads and mark the shorter junction parts as soft-clip" why are shorter junction parts marked as soft-clip? How will you know which parts of real reads are short and should be marked as soft-clip?

Thank you for your comments, we edited the text to reducing the confusing points you mentioned.

8. There is a lack of details about filtering the alignment results (page 7 lines 7-8): sequencing quality, mapping quality and mismatch rates should be described better with specific parameters for every step.

Thank you for your comments, we revised the text based on your suggestion.

9. There is lack of details regarding clustering procedure of reads surrounding or containing breakpoints. The clustering procedure (cluster extension) could be supported by a scheme/figure for better understanding. Also, which reads will you cluster in real data when you do not know which of them contain breakpoints? Reads which are not aligned to the reference human genome? This should be described in the text.

Thank you for your comments, we edited the text based on your suggestion.

10. Section Assembling could be accompanied by a better scheme/figure or more text for the author's restore algorithm. Figure 2 does not clearly explain how the restore algorithm is restoring the bisulfite-altered sequence to the original and more details are needed. For example, which strain on Figure 2 is original and which is restored.

Thank you for your comments, we revised this part to make the method clearer.

11. Also, are there any studies where such an approach for assembling (as author's restore algorithm) was previously used? References should be provided or it should be mentioned if it is completely novel approach.

Thank you for your comments, we edited the text to make the new approach clearer.

12. Last part of the "Methods" suggests alignment of unmapped to the human reference genome reads to the viral reference sequence. In real data when you do not know what types of viruses are contained/integrated in the analyzed sample which viral references should the user use? Should it be all known viral reference sequences? Or should the user perform an initial analysis for identification of virus(es) in the sample and then use this pipeline only for detection of breakpoints (as in VirusSeq)?

We performed WGS and WGBS for PLC/PRF/5 cell line and anaylizer the data. The result is showed in Table 1.

13. Figure 3 needs more description in text of what exactly it shows, and a clearer explanation in the legend. I do not see how Figure 3 demonstrates the extraction of the virus fragment location from the alignment result.

Thank you for your comments, we edited the text based on your suggestion.

Minor Concerns
1.Manuscript pages and formulas must be numbered.

Powered by Editorial Manager® and ProduXion Manager® from Aries Systems Corporation

Thank you for your comments, we numbered the text based on your suggestion.

2.There are discrepancies in the text regarding what chromosome was used for simulation: chr 18 on page 6 line 6 and chr 1 on page 10 line 13. In the section "Data description in silico" authors mentioned that simulation of breakpoints was performed only on PE reads (90 bp), but in Table 1 and in Discussion they are mentioned simulation of PE 50, 90, 150. Authors should coordinate through all sections of the article - what and how they performed analysis and simulation in this study.

Thank you for your comments, we edited the text based on your suggestion.

3.In the section Assembling "Q" should be defined in second formula (page 8, line 12).

Thank you for your comments, we edited the text based on your suggestion.

4.In "Discussion" (page 9, line 12) a reference should be provided for the statement "Virus usually integrates into regions that homologous to both human and virus (micro-homologous)".

Thank you for your comments, we edited the text based on your suggestion.

5.On page 9 lines 14-15 authors claim "The accuracy of predicted breakpoints can reach over 70%" and then on page 10, line 1 "Bs-virus-finder is capable to find more than 80% of virus integration with the accuracy more than 90%". Should be consistent in description of simulation's results.

Thank you for your comments, we revised the text based on your suggestion. Particularly, as the result showed in Table S4, for input sequence that the length around 50bp, BS-virus-finder is capable to find the virus integration with the accuracy more than 70%; for the input sequence between 90bp and 150bp, BS-virus-finder is capable to find the virus integration with the accuracy more than 90%.

6.There are many English grammar errors through the text which should be corrected. For example, stimulated instead of simulated. Also, in sentence "Generally, however, bwa-meth [13] performed very well. It indicated virus breakpoints might be hardly found by our BS virus finder" if breakpoints could be hardly found, why was this manuscript written?

We have deleted this confusing describing.

7.Paragraph on page 6, lines 15-19 not suits to Result section and should be moved to Introduction, for example.

Thank you for your comments, we revised the text based on your suggestion.

Reviewer #2: The study presented by Gao and colleagues discusses a software, BS-virus-finder, which allows the detection of viral integration breakpoints in human genomes using bisulfite sequencing data. Importantly, this appears to be the first software which allows the detection of viral integration breakpoints from bisulfite sequencing data.

1)Introduction: Define the abbreviations 'SNP', 'DMR' and 'ASM'.

Thank you for your comments, we edited the text based on your suggestion.

2)Introduction: Abbreviations need to be harmonised: The abbreviation for 'whole-genome bisulfite sequencing' is given as 'Bis-seq' and 'WGBS'.

Thank you for your comments, we edited the text based on your suggestion.

3) Introduction: The software SMAP appears to be referenced as reference [11] as well as reference [1].

	Thank you for your comments, we edited the text based on your suggestion.<br><br>4) Data description in silico: I don't understand the following sentences: 'Generally, however, bwa-meth [13] performed very well. It indicated virus breakpoints might be hardly found by our BS virus finder.' - Does this mean that the performance of the bwa-meth software alone is superior to the presented BS-virus-finder software which is based on bwa-meth? Please clarify.<br><br>Thank you for your comments, we edited the text based on your suggestion. We deleted the confusing description.<br><br>5) The authors should provide a table where they compare the BS-virus-finder software with other software used for the detection of viral integration breakpoints, such as VirusFinder (PMID: 23717618), VERSE (PMID: 25699093), Virus-Clip (PMID: 26087185), Vy-PER (PMID: 26166306), Seeksv (PMID: 27634948) or any other software of relevance.<br><br>We performed WGS and WGBS for PLC/PRF/5 cell line. The result was analyzed by Vy-per, virus-clip(REF) and Virus Finder2, respectively. These results were compared with WGBS result analyzed by BSVF. The comparison of the result is showed in Table 1.<br><br>6) It would be good if the authors could provide an example/examples where they show the performance of the BS-virus-finder on 'real' datasets (perhaps datasets which have been analysed by using other software tools?).<br><br>We performed WGS and WGBS for PLC/PRF/5 cell line. The result were analyzed by Vy-per, virus-clip and Virus Finder2, respectively. These results were compared with WGBS result analyzed by BSVF. The comparison of the result is showed in Table 1.<br><br>7) Figure 2/Legend figure 2: The 'G' in a 'CG' shows for the 'Crick' strand the 'm' in subscript to indicate that this is a methylation-modified base. However, this is confusing as it leaves the impression that the 'G' is methylated instead of the corresponding 'C'.<br><br>Methylation actually occurs at C, however when C in Crick strand transformed into T, at its reverse complementary strand it is G transformed into A. Therefore, Gm equals Cm at Crick strand.
**Additional Information:**	
**Question**	**Response**
Are you submitting this manuscript to a special series or article collection?	No
**Experimental design and statistics**<br><br>Full details of the experimental design and statistical methods used should be given in the Methods section, as detailed in our Minimum Standards Reporting Checklist. Information essential to interpreting the data presented should be made available in the figure legends.<br><br>Have you included all the information requested in your manuscript?	Yes
**Resources**	Yes

A description of all resources used, including antibodies, cell lines, animals and software tools, with enough information to allow them to be uniquely identified, should be included in the Methods section. Authors are strongly encouraged to cite Research Resource Identifiers (RRIDs) for antibodies, model organisms and tools, where possible.<br><br>Have you included the information requested as detailed in our Minimum Standards Reporting Checklist?	
**Availability of data and materials**<br><br>All datasets and code on which the conclusions of the paper rely must be either included in your submission or deposited in publicly available repositories (where available and ethically appropriate), referencing such data using a unique identifier in the references and in the "Availability of Data and Materials" section of your manuscript.<br><br>Have you have met the above requirement as detailed in our Minimum Standards Reporting Checklist?	Yes

Manuscript Click here to download Manuscript Manuscript0905.doc

BS-virus-finder: virus integration calling using bisulfite-sequencing data

Shengjie Gao[1,2,7,8,9*], Xuesong Hu[2*], Changduo Gao[3*], Kai Xiong[4*], Fengping Xu[5,10,13*], Xiao Zhao[2,11], Haixiao Chen[5,13], Shancen Zhao[5,7], Mengyao Wang[5], Dongke Fu[2], Xiaohui Zhao[6], Jie Bai[5], Bo Li[2], Song Wu[8], Shengbin Li[2,12#], Huangming Yang[5, 7,11#], Lars Bolund[9#], Christian N. S. Pedersen[1#]

[1] Bioinformatics Research Centre, Aarhus University, Aarhus, Denmark
[2] BGI-Forensic, Shenzhen 518083, China
[3] College of Computer Science & Technology, Qingdao University, Qingdao 266071, China
[4] Department of Veterinary Clinical and Animal Sciences, University of Copenhagen, Copenhagen, Denmark
[5] BGI-Shenzhen, Shenzhen 518083, China
[6] College of Mathematics & Statistics, Changsha University of Science & Technology, Changsha 410114, China
[7] James D. Watson Institute of Genome Sciences, Hangzhou 310058, China
[8] The Affiliated Luohu Hospital of Shenzhen University, Shenzhen University, Shenzhen 518000, China.
[9] Department of Biomedicine, Aarhus University, Aarhus, Denmark
[10] Department of Biology, University of Copenhagen, Copenhagen, Denmark
[11] BGI Education Center, University of Chinese Academy of Sciences.
[12] Shenzhen Key Laboratory of Forensics, BGI-Shenzhen, Shenzhen 518083, China.
[13] China National GeneBank, BGI-Shenzhen, Shenzhen 518120, China

[*] These authors contributed equally to this work

[#] These authors equally directed the work

Abstract:

Background: DNA methylation plays a key role in regulating gene expression and carcinogenesis. Bisulfite sequencing studies mainly focus on calling SNP, DMR, and ASM. Until now, only a few software tools focus on virus integration using bisulfite sequencing data.

Findings: We have developed a new and easy-to-use software tool, named as BS-virus-finder (https://github.com/BGI-SZ/BSVF), to detect viral integration breakpoints in whole human genomes.

Conclusions: BS-virus-finder demonstrates high sensitivity and specificity, and is useful in epigenetic studies and to reveal the relationship between viral integration and DNA methylation. BS-virus-finder is the first software tool to detect virus by using bisulfite sequencing data.

Keyword: Virus integration, Bisulfite sequencing, Carcinogenesis

Findings

Introduction

DNA methylation plays crucial roles in many areas including development [1, 2] and X chromosome inactivation [3] by regulating genetic imprinting and epigenetic modification without altering DNA sequences. Previous studies have showed strong association of DNA methylation with cancer. The methylation status altering related carcinogenesis [4], cancer recurrence [5] and metastasis [6] has already been revealed by emerging bisulfite sequencing (BS) technology. BS technology can investigate DNA methylation changes with single-base accuracy. Treatment of DNA with bisulfite converts unmethylated cytosine residues to uracil, but leaves 5-methylcytosine residues unmodified [7]. Thus, bisulfite treatment introduces specific changes in the DNA sequence that depend on the methylation status of individual cytosine residues, yielding single-nucleotide resolution information about the methylation status of a segment of DNA (Figure 1). Various analyses can be performed on the altered sequences to retrieve this information. BS technology can reveal differences between cytosines and thymidine and sequence change resulting from bisulfite conversion. For the bases without methylation, all C will change to T on both strands. After directional library preparation, we have two different conversions: The Watson, and the Crick strand, as show in Figure 1. On the Watson strand, methylated C remains C, and un-methylated C changes to T. On the Crick strand, the reverse complement happens, i.e. methylated C remains C but in

sequenced reads it is reverse complement to G, and un-methylated C changes to T, leading to the reverse complement base A in sequenced reads. Since base C can either be methylated or un- methylated, we can use IUPAC nucleotide code “Y” and “R” to represent C/T and G/A respectively. So, after bisulfite treatment, base C changes to Y on Watson strand, and base G changes to R on Crick strand.

Whole-genome based bisulfite sequencing (WGBS) has been developed to detect DNA methylation. A recent clinical study showed that DNA methylation is associated with viral integration [8, 9]. Whole-genome BS (WGBS) data can be analyzed to investigate the sequence mapping and alignment via BSMAP [10], Bismark [11] and bwa-meth [12], to detect DMR (different methylation regions)via software QDMR [13], DMAP [14] and SMAP [11], to identify SNP (single sucleotide polymorphism) via software BS-SNPer [15] and Bis-SNP [16], and finding ASM (allele-specific DNA methylation) via SMAP [17], Methy-Pipe [18]. However, none of them can be used for virus integration loci calling, and no software tool is currently available to detect virus integration loci by analyzing BS data. Therefore, we have developed a software tool to detect the virus integration loci by genome-wide BS analysis.

Description in silico and real data.

Different types of PE (paired-end) reads (50bp, 90bp, 150bp) that include 700 breakpoints in chromosome 18 (chr. 18) of GRCh38 were simulated in our study. Input fragments of 50 to 400 bp were randomly selected from chromosome 18 in the

GRCh37 assembly (hg19) of the human genome. The length of viral integration was between 45 bp to 180 bp. After the alignment, the mapping accuracy of each of the 17 different types of reads mapping was calculated (Figure 2). Mapping accuracy varied among the 17 types of read mappings in our simulation (Figure S1, S2, S3). In summary, the accuracies of several kinds of the reads mappings were low (Table S1, S2, S3), which may raise false-negative rate. Generally, however, bwa-meth [12] performed very well.

Bisulfite sequencing is a marvelous and sophisticated technique to study DNA cytosine methylation. Bisulfite treatment followed by PCR amplification specifically converts methylated cytosine to thymine. By cooperating with next generation sequencing technology, it is able to detect the methylation status of every cytosine in the whole genome. Moreover, longer reads make it possible to achieve higher accuracy. Besides simulated data, the PLC/PRF/5 hepatocellular carcinoma cell lines were from American Type Culture Collection (ATCC, Manassas, VA) were cultured as previously described [19]. The cell line was validated by STR makers (Figure S4). We performed WGS and WGBS sequencing of this cell line, the result is showed in Table S4. Table 1 shows the analysis result for WGS data, which was compared with the output results analyzed by Vy-per [20], virus-clip[21] and Virus Finder2 [22].

Method for calling virus integration

The reads coverage situation for one integration is shown in Figure 3. Four steps were implemented to detect virus integration:

1. Alignment

We use bwa-meth [12] to align bisulfite treated sequencing reads to a hybrid reference that contains both human genome and virus sequences. For chimeric reads from the junction parts, BWA-MEM [23] will align it to one organism and mark the unmapped part as soft clipping, which is in fact from the other organism. This enables us to find breakpoints directly from the alignment.

2. Clustering

After alignment, the result was filtered. We select read pairs with one read match by the following criterion: the Phred-scaled mapping quality is bigger than 30 (>=30), and at least one soft clipping is longer than 5 bp (>=5). The mapped parts of reads, which is marked as "M" by its CIGAR string, are covering the human reference genome. For paired reads, we also add the gap between two mapped reads to their covered region, making read 1 and read 2 be continuous covered on human reference. Each continuous region with at least 1 bp overlap are defined as a cluster. All reads involved are selected to form the cluster. The soft clippings that remain are viral junction candidates. Read pairs with one read mapped on virus also indicate potential virus junction between the read pair.

3. Assembling

Within one cluster, all soft clipping start sites are collected. The position with the most abundance of start sites is identified as the most likely candidate breakpoint. All its clipping sequences are extracted and aligned together. A restore algorithm was used to calculate the most possible base in each position based on the aligned bases and its sequencing quality. The

algorithm is based on a Bayesian model, where we compute the posteriori probability estimation for A, C, G, T as:

$$\begin{aligned} P(T_i \mid D) &= \frac{P(T_{Wi})P(D \mid T_{Wi})}{\sum_{x=1}^{S} P(T_{Wx})P(D \mid T_{Wx})} \times \frac{P(T_{Ci})P(D \mid T_{Ci})}{\sum_{x=1}^{S} P(T_{Cx})P(D \mid T_{Cx})} \\ &= C_0 \times P(D \mid T_{Wi}) \times P(D \mid T_{Ci}) \\ C_0 &= \frac{P(T_{Wi})}{\sum_{x=1}^{S} P(T_{Wx})P(D \mid T_{Wx})} \times \frac{P(T_{Ci})}{\sum_{x=1}^{S} P(T_{Cx})P(D \mid T_{Cx})} \end{aligned} \quad (1)$$

Here, D is the observation of the NGS reads on given position. P(Ti|D) is the likelihood component, which can be interpreted as the probability of observing D when the true genotype is T_i. D_W be a realization (or observation) of the NGS reads in the Watson strand. D_C be a realization (or observation) of the NGS reads in Crick strand. $P(T_{Wi}|D)$ is the likelihood component, which can be interpreted as the probability of observing D when the true genotype is T_{Wi}. $P(T_{Ci}|D)$ is the likelihood component, which can be interpreted as the probability of observing D when the true genotype is T_{Ci}. At each virus location, prior probability P(Ti) of each genotype Ti was set according to the Table S5. The likelihood P(D|Ti) for the assumed genotype Ti was calculated from the observed allele types in the sequencing reads in formula 2. Thus, on the Watson strand it is $P(D_W|T_i)$, on the Crick strand it is $P(D_C|T_i)$. We defined the likelihood of observing allele d_k in a read for a possible haploid genotype T as $P(d_k|T)$, and on the Watson strand it is $P(d_{Wk}|T)$, and on the Crick strand it is $P(d_{Ck}|T)$. So, for a set of n observed alleles at a locus, $D = \{d_1, d_2, \ldots, d_n\}$ on each strand, these probabilities are computed as shown by formula 3 & 4, where Q stands for the base quality from the fastaq file.

$$P(D_W \mid T_i) = \prod_{k=1}^{m} P(d_{Wk} \mid T), P(D_C \mid T_i) = \prod_{k=1}^{n} P(d_{Ck} \mid T). \quad (2)$$

$$P(d_{Wk} \mid T) = \begin{cases} 1 - 10^{-\frac{Q}{10}} & (T \in \{A, C, G\}) \\ \dfrac{1 - 10^{-\frac{Q}{10}}}{2} & (T \in \{T\}) \end{cases}, \quad (3)$$

$$P(d_{Ck} \mid T) = \begin{cases} 1 - 10^{-\frac{Q}{10}} & (T \in \{C, G, T\}) \\ \dfrac{1 - 10^{-\frac{Q}{10}}}{2} & (T \in \{A\}) \end{cases}.$$

We used “Y” and “R” to represent C/T and G/A respectively (IUPAC nucleotide code). If a region is covered by both the Watson strand and the Crick strand, we were able to deduce the original base from Y or R by calculation.

4. Detection of viral integrations

The assembled clipping regions above were mapped to the given virus reference sequence with a Smith-Waterman local alignment tool from EMBOSS package [24], which support IUPAC DNA codes Y and R. Virus fragment location is extracted from the alignment results.

Discussion

In summary, we have implemented the first software tool to detect virus integration using BS data. Our software is based on bwa-meth, and by assembling and aligning soft-clip regions, it can find the virus breakpoints. However, accuracy of reads surrounding the breakpoints needs to be further improved. Virus usually integrates into regions that are homologous to both human and virus (micro-homologous) [25]. Therefore, we consider the breakpoints predicted by our software tool that are within 10 bp of a real breakpoint as being correctly identified (Figure S2). With this definition, the accuracy of our predicted breakpoints can reach over 70%. Our results will be useful for analyzing BS data and related applications. Some of the results come with only the location on human genome, and has the virus location missing. This may be due to the shortage of virus fragments. We stimulated three kinds of reads, PE50,

90, and 50 with various lengths, and further stimulated virus-inserted fragment with different length as well (Table S6), thus all cases described in Figure 2 are mimicked here. As the result in Table S6 showed, the longer the reads, the more accurate the prediction can be achieved. In particularly, for read lengths around 50 bp, BS-virus-finder is capable to find the virus integration with an accuracy of more than 70%; for the read lengths between 90bp and 150bp, BS-virus-finder is capable to find the virus integration with an accuracy of more than 90%. Besides simulated data, we have performed WGS and WGBS sequencing of the PLC/PRF/5 hepatocellular carcinoma cell line (Table S4). As the results showed, when the length of input is large than 150bp, the analysis result of WGBS is similar to the one of WGS. Additionally, BS-virus-finder is able to find breakpoints in 8 out of 9 regions which are identified by FISH [8]. Based on these experimental results, we believe that BS-virus-finder is a powerful software tool to analyze virus-integration using BS data.

Availability and requirements

Project Name: BS-virus-finder: virus integration calling using bisulfite-sequencing data

Project home page: https://github.com/BGI-SZ/BSVF

Operating system: Linux

Programming language: Perl, Python, C

License: GPL v3

Availability of supporting data

Data used in this paper is simulated based on random insertion of HBV sequence to human

chromosome 1 sequence. A Perl script named "simVirusInserts.pl" is included, and our simulation schema is coded within. We have run the simulation several times and the result shows no significant difference. The PLC/PRF/5 hepatocellular carcinoma cell lines were from American Type Culture Collection (ATCC, Manassas, VA) and sequenced by HiSeq X Ten System from Novogene company. WGS and WGBA data have been submitted to NCBI SRA project PRJNA400455.

Competing interests

The authors declare that they have no competing interests.

Acknowledgements

We appreciate the supporting of Xiaolin Liang and Hengtong Li in College of Mathematics & Statistics, Changsha University of Science & Technology, for their contributing advice to our research. This work was funded by the National Natural Science Foundation of China (81602477) and Shenzhen Municipal Government of China (ZDSYS201507301424148).

References:

1. Wang Y, Shang Y: **Epigenetic control of epithelial-to-mesenchymal transition and cancer metastasis**. *Experimental cell research* 2013, **319**(2):160-169.
2. O'Doherty AM, Magee DA, O'Shea LC, Forde N, Beltman ME, Mamo S, Fair T: **DNA methylation dynamics at imprinted genes during bovine pre-implantation embryo development**. *BMC developmental biology* 2015, **15**:13.
3. Cotton AM, Price EM, Jones MJ, Balaton BP, Kobor MS, Brown CJ: **Landscape of DNA methylation on the X chromosome reflects CpG density, functional chromatin state and X-chromosome inactivation**. *Human molecular genetics* 2015, **24**(6):1528-1539.
4. Kamdar SN, Ho LT, Kron KJ, Isserlin R, van der Kwast T, Zlotta AR, Fleshner NE, Bader G, Bapat B: **Dynamic interplay between locus-specific DNA methylation and hydroxymethylation regulates distinct biological pathways in prostate**

carcinogenesis. *Clinical epigenetics* 2016, **8**:32.

5. Haldrup C, Mundbjerg K, Vestergaard EM, Lamy P, Wild P, Schulz WA, Arsov C, Visakorpi T, Borre M, Hoyer S *et al*: **DNA methylation signatures for prediction of biochemical recurrence after radical prostatectomy of clinically localized prostate cancer**. *Journal of clinical oncology : official journal of the American Society of Clinical Oncology* 2013, **31**(26):3250-3258.
6. Kim JH, Dhanasekaran SM, Prensner JR, Cao X, Robinson D, Kalyana-Sundaram S, Huang C, Shankar S, Jing X, Iyer M *et al*: **Deep sequencing reveals distinct patterns of DNA methylation in prostate cancer**. *Genome research* 2011, **21**(7):1028-1041.
7. Darst RP, Pardo CE, Ai L, Brown KD, Kladde MP: **Bisulfite sequencing of DNA**. *Current protocols in molecular biology / edited by Frederick M Ausubel [et al]* 2010, **Chapter 7**:Unit 7 9 1-17.
8. Watanabe Y, Yamamoto H, Oikawa R, Toyota M, Yamamoto M, Kokudo N, Tanaka S, Arii S, Yotsuyanagi H, Koike K *et al*: **DNA methylation at hepatitis B viral integrants is associated with methylation at flanking human genomic sequences**. *Genome research* 2015, **25**(3):328-337.
9. Lillsunde Larsson G, Helenius G, Sorbe B, Karlsson MG: **Viral load, integration and methylation of E2BS3 and 4 in human papilloma virus (HPV) 16-positive vaginal and vulvar carcinomas**. *PloS one* 2014, **9**(11):e112839.
10. Xi Y, Li W: **BSMAP: whole genome bisulfite sequence MAPping program**. *BMC bioinformatics* 2009, **10**:232.
11. Krueger F, Andrews SR: **Bismark: a flexible aligner and methylation caller for Bisulfite-Seq applications**. *Bioinformatics* 2011, **27**(11):1571-1572.
12. Pedersen BS EK, De S, Yang IV, Schwartz DA: **Fast and accurate alignment of long bisulfite-seq reads**. *eprint arXiv* 2014.
13. Zhang Y, Liu H, Lv J, Xiao X, Zhu J, Liu X, Su J, Li X, Wu Q, Wang F *et al*: **QDMR: a quantitative method for identification of differentially methylated regions by entropy**. *Nucleic acids research* 2011, **39**(9):e58.
14. Stockwell PA, Chatterjee A, Rodger EJ, Morison IM: **DMAP: differential methylation analysis package for RRBS and WGBS data**. *Bioinformatics* 2014.
15. Gao S, Zou D, Mao L, Liu H, Song P, Chen Y, Zhao S, Gao C, Li X, Gao Z *et al*: **BS-SNPer: SNP calling in bisulfite-seq data**. *Bioinformatics* 2015, **31**(24):4006-4008.
16. Liu Y, Siegmund KD, Laird PW, Berman BP: **Bis-SNP: Combined DNA methylation and SNP calling for Bisulfite-seq data**. *Genome biology* 2012, **13**(7):R61.
17. Gao S, Zou D, Mao L, Zhou Q, Jia W, Huang Y, Zhao S, Chen G, Wu S, Li D *et al*: **SMAP: a streamlined methylation analysis pipeline for bisulfite sequencing**. *GigaScience* 2015, **4**:29.
18. Jiang P, Sun K, Lun FM, Guo AM, Wang H, Chan KC, Chiu RW, Lo YM, Sun H: **Methy-Pipe: an integrated bioinformatics pipeline for whole genome bisulfite sequencing data analysis**. *PloS one* 2014, **9**(6):e100360.
19. Carr BI, Cavallini A, Lippolis C, D'Alessandro R, Messa C, Refolo MG, Tafaro A:

Fluoro-Sorafenib (Regorafenib) effects on hepatoma cells: growth inhibition, quiescence, and recovery. *J Cell Physiol* 2013, **228**(2):292-297.

20. Forster M, Szymczak S, Ellinghaus D, Hemmrich G, Ruhlemann M, Kraemer L, Mucha S, Wienbrandt L, Stanulla M, Group UFOSCwI-BS *et al*: **Vy-PER: eliminating false positive detection of virus integration events in next generation sequencing data**. *Sci Rep* 2015, **5**:11534.
21. Ho DW, Sze KM, Ng IO: **Virus-Clip: a fast and memory-efficient viral integration site detection tool at single-base resolution with annotation capability**. *Oncotarget* 2015, **6**(25):20959-20963.
22. Wang Q, Jia P, Zhao Z: **VERSE: a novel approach to detect virus integration in host genomes through reference genome customization**. *Genome Med* 2015, **7**(1):2.
23. Li H: **Aligning sequence reads, clone sequences and assembly contigs with BWA-MEM**. *eprint arXiv* 2013:3.
24. Rice P, Longden I, Bleasby A: **EMBOSS: the European Molecular Biology Open Software Suite**. *Trends in genetics : TIG* 2000, **16**(6):276-277.
25. Hu Z, Zhu D, Wang W, Li W, Jia W, Zeng X, Ding W, Yu L, Wang X, Wang L *et al*: **Genome-wide profiling of HPV integration in cervical cancer identifies clustered genomic hot spots and a potential microhomology-mediated integration mechanism**. *Nature genetics* 2015, **47**(2):158-163.

Table 1. The comparison of BS-virus-finder with other software using real data.

Chr	BSVF			Vy-per			virus-clip			Virus Finder2		
	HB	VB	VE	HB	VB	VE	HB	VB	VE	HB	VB	VE
chr1	143272758	2945	3102									
chr2	-			-			52018758	207	281			
chr3*	131451702	1212	1322	-			131451701	1282	1403	131451701	1405	
chr3*	131453124	1416	1515	-			131453353	1416	1538			
chr4*	180586417	136	378							180586416	59	
chr4*	180587608	394	594	180586607	167	231	180587608	500	632	180587607	634	
chr5*	1297478	1174	1315	-			1297478	1241	1385	1297477	1388	
chr7	110894616	2739	2748									
chr8*	35446393			35446214	2402	2455	35446601	2390	2519	35446392	2396	2608
chr8	-						106944290	698	1077			
chr11*	65040943	2631	2767	-			-			65040964	2532	
chr12*	109573899	721	815	109573677	668	734	109573899	705	815			
chr13	33088123	1521	1603	-			-					
chr13	33088561	1917	2066	-			33088561	1995	2133	33088560	2133	
chr16*	69947046	2055	2826									
chr16*	70169959	2055	2735							70169971	2064	2240
chr16	74425602	2062	2665									
chr17*	82105786	407	489	82105984	368	435	82105783	347	489			
chr17*	82107626	2177	2321	-			82107710	2048	2159	82107625	2045	
chr19	41783064	687	804	-			41782971	761	905			
chr20	20473566	2415	2565									

BSVF used WGBS data, and other software used WGS data.

* supported by previous FISH experiments [8].

HB: Host breakpoint.

VB: Virus Begin is the revealed left most position on virus.

VE: Virus End is the right most position on virus.

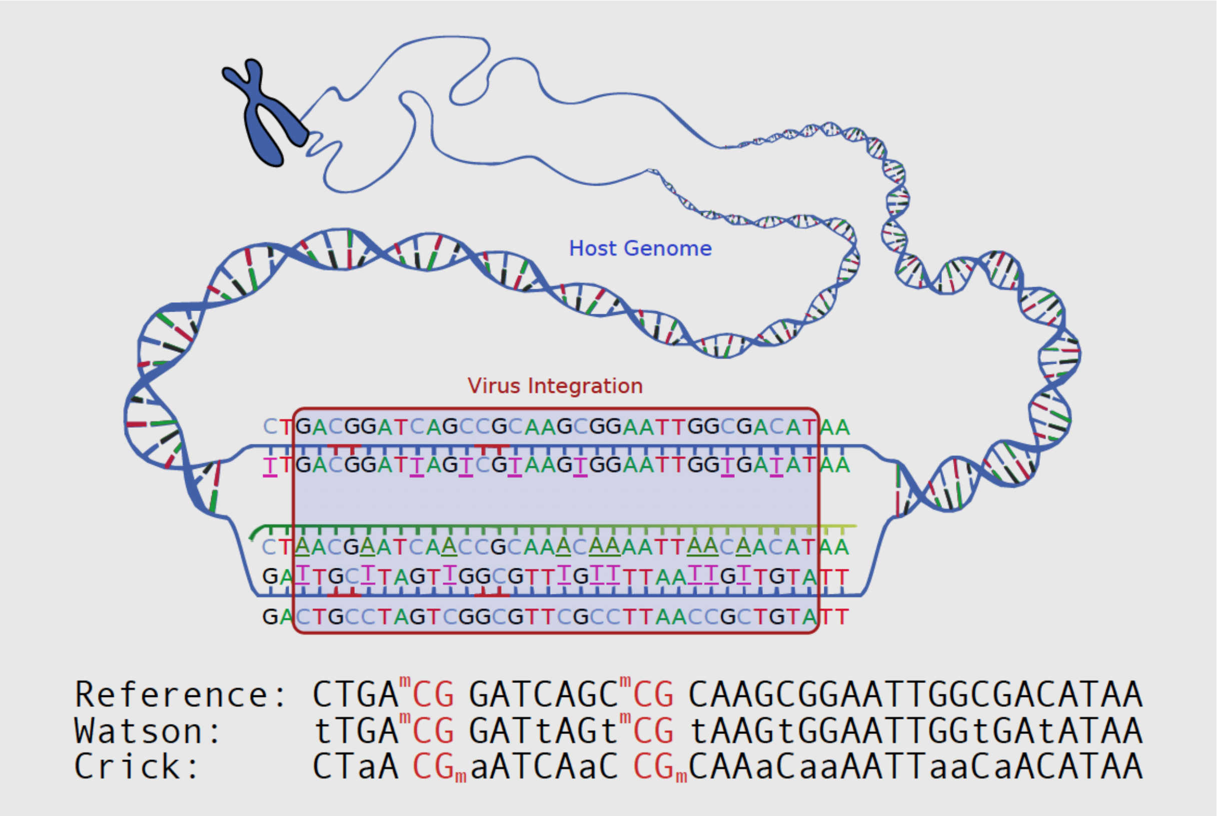

Figure 1. The illustration of bisulfite-altered sequence to the original.

Reference is the original sequence prior to bisulfite treatment. After directional library preparation, we have two different conversion: Watson and Crick strand.

Methylation sites were showed as read bases. Bisulfite treated base may alter the original base from C to T. m indicated methylation-modified base. Low-case letter indicates the bisulfite-altered base.
As we can see, half of the probability of each T is C in Watson strand and half of the probability of each A is G in Crick strand.

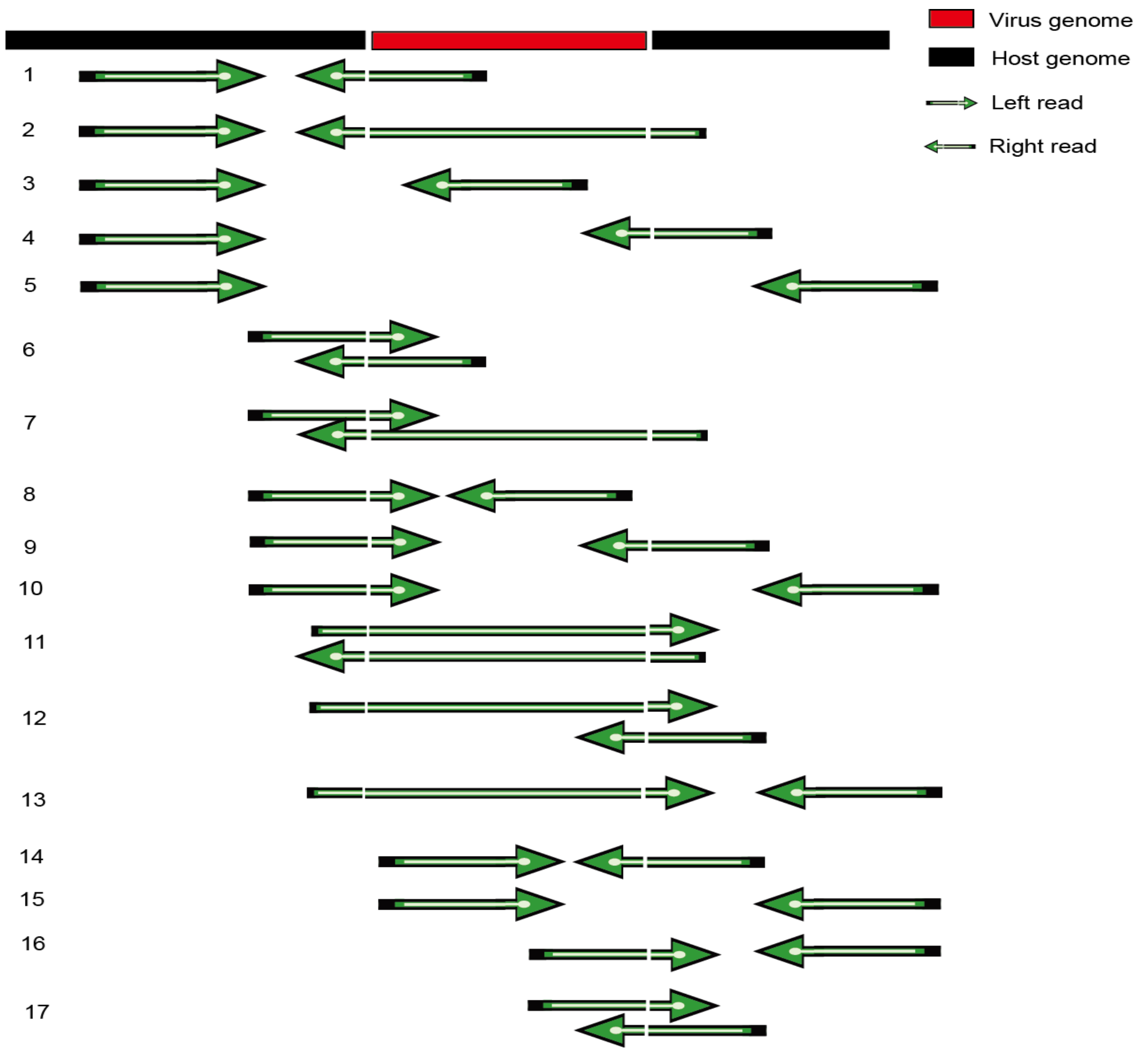

Figure 2. Principal types of mapping reads around the viral integration site.

Red bar, the virus sequence inserted in host genome; Green arrow, mapping reads with different directions; Breakpoints indicate logical division between host genome and virus, which are physically linked.

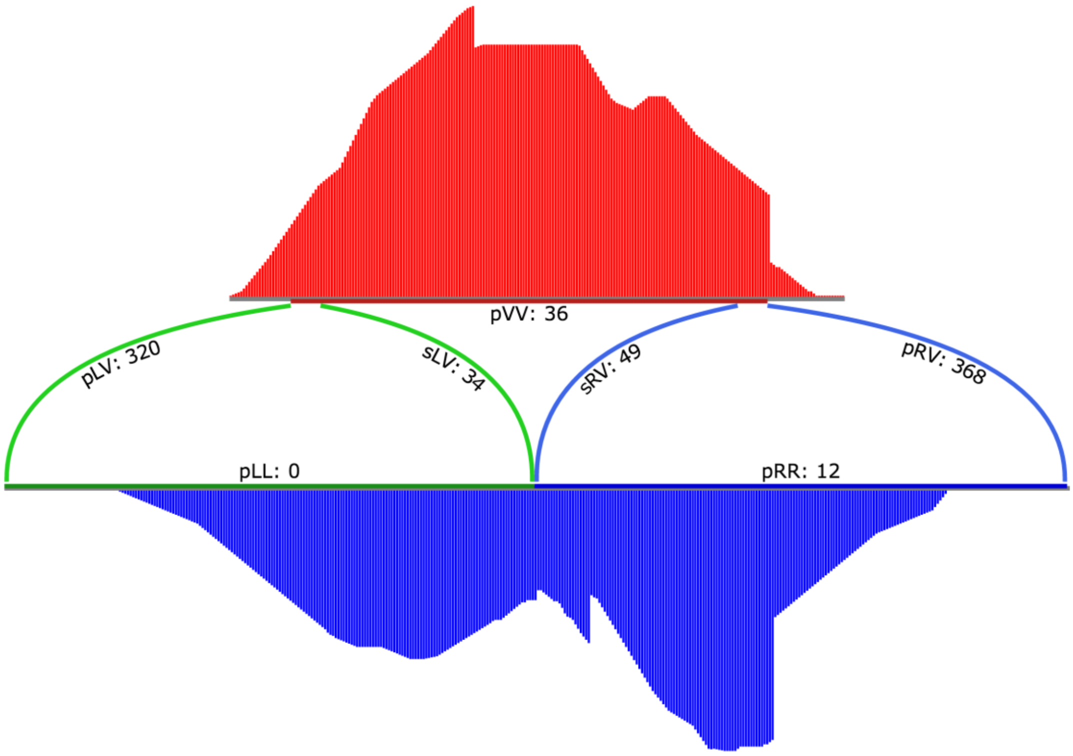

Figure 3. A demo plot of one viral integration cluster in its pre-insertion form.
Plot was randomly selected from simulated breakpoints.
The red virus fragment(V) above will be inserted into the center point of the green(L) and blue(R) human fragment below to form the sequenced sample. Bars show the coverage depth of sequencing reads.
Curves represent the linkage events supported by pair-end sequencing reads, and the number besides shows the read count.

Click here to access/download
Supplementary Material
Supplementary-bsfinder.docx

Personal Cover
Click here to download Personal Cover Personal Cover.docx

AARHUS
UNIVERSITY
BIOINFORMATICS RESEARCH CENTRE (BIRC)

GigaScience Editorial Office

Resubmission of paper

Dear Editor in Chief

Thank you very much for the opportunity to resubmit our paper "BS-virus-finder: virus integration calling using bisulfite-sequencing data" (GIGA-D-17-00032) cf. your e-mail from June 6, 2017. We have addressed the points raised by the reviewers to the best of our abilities, and as outlined in our response to reviewers. We hope that you find everything in order.

Kind regards

Christian Nørgaard Storm Pedersen
(on behalf of all the authors of the paper)

Bioinformatics
Centre (BiRC)

Christian Nørga
Pedersen

Centre director
professor

Date: 05 Septe

Direct Tel.: +45
Mobile Tel.: +45
E-mail: cstorm@

Web: au.dk/en

Sender's CVR n
