# Supplementary material for: BS-virus-finder: virus integration calling using bisulfite sequencing data
Source: Gigascience. 2017 Dec 18;7(1):1–7. doi: 10.1093/gigascience/gix123 (PMC5788064; doi:10.1093/gigascience/gix123)
Supplement: GIGA-D-17-00032_Revision_2.pdf [file gix123_giga-d-17-00032_revision_2.pdf]

GigaScience

BS-virus-finder: virus integration calling using bisulfite-sequencing data

--Manuscript Draft--

**Manuscript Number:**	GIGA-D-17-00032R2
**Full Title:**	BS-virus-finder: virus integration calling using bisulfite-sequencing data
**Article Type:**	Technical Note
**Funding Information:**	Young Scientists Fund of the National Natural Science Foundation of China (81602477) — Dr. Shengjie Gao<br>Shenzhen Municipal Government of China (ZDSYS201507301424148) — Prof. Shengbin Li
**Abstract:**	Background: DNA methylation plays a key role in regulating gene expression and carcinogenesis. Extant methylation bisulfite sequencing (BS) researches mainly focus on calling SNP, DMR, and ASM, instead of virus integration positions.<br>Findings: We developed a new and easy-to-use software, named as BS-virus-finder (https://github.com/BioInfoTools/BSVF), to detect viral integration breakpoints in whole human genomes.<br>Conclusions: BS-virus-finder demonstrates moderate sensitivity and specificity, and is useful to be applied in epigenetic researches and to reveal the relationship between viral integration and DNA methylation. BS-virus-finder is the first software to detect virus by using bisulfite sequencing data.
**Corresponding Author:**	Christian Pedersen<br><br>DENMARK
**Corresponding Author Secondary Information:**	
**Corresponding Author's Institution:**	
**Corresponding Author's Secondary Institution:**	
**First Author:**	Shengjie Gao
**First Author Secondary Information:**	
**Order of Authors:**	Shengjie Gao
	Xuesong Hu
	Changduo Gao
	Kai Xiong
	Fengping Xu
	Xiao Zhao
	Haixiao Chen
	Shancen Zhao
	Mengyao Wang
	Dongke Fu
	Xiaohui Zhao
	Jie Bai
	Likai Mao
	Bo Li
	Song Wu

Powered by Editorial Manager® and ProduXion Manager® from Aries Systems Corporation

	Jian Wang
	Shengbin Li
	Huanming Yang
	Lars Bolund
	Christian Pedersen
**Order of Authors Secondary Information:**	
**Response to Reviewers:**	Reviewer reports:<br>Reviewer #1: Re-review on manuscript<br>BS-virus-finder: virus integration calling using bisulfite-sequencing data<br>Shengjie Gao, Ph.D; Xuesong Hu; Changduo Gao; Kai Xiong; Fengping Xu; Xiao Zhao; Haixiao Chen; Shancen Zhao; Mengyao Wang; Dongke Fu; Xiaohui Zhao; Jie Bai; Bo Li; Song Wu; Shengbin Li; Huanming Yang; Lars Bolund; Christian Pedersen.<br>by Lada A Koneva, PhD<br><br>Introduction<br>The reviewed manuscript (BS-virus-finder: virus integration calling using bisulfite-sequencing data) describes developed software to detect viral integration breakpoints in whole-genome bisulfite-sequencing data (WGBS).<br>The manuscript was improved according to my suggested concerns and questions, but there are still some questions.<br><br>Questions<br>As a consequence of choosing HBV for simulation and PLC/PRF/5 hepatocellular carcinoma cell lines (which harboring HBV) as a real data, there is still unanswered my question about how this algorithm will work in case of HPV integration. There are about 170 types of HPV, with high commonality between different types, and about 15 types are carcinogenic. How authors suggest creating a "hybrid reference that contains both human genome and virus sequences" in case of HPV? Which viral types could be chosen for alignment of assembled clipping regions in case of HPV contamination? Probably this algorithm could not accurately predict which type(s) of HPV contaminates the sample. It would be useful if authors could provide their thoughts if this algorithm also will work in case of virus like HPV or this should be restricted to analysis of HBV integration only.<br><br>Although most programs input only one virus sequence, BSVF users are allowed to set their own multiple virus sequences. For each custom virus genome, BSVF constructs a hybrid reference by combining the virus sequences and human reference. For viruses with large number of strains, such as HPV, to get more meaningful results, we suggest users select some representative sequences from highly similar (such as >90% similarity) sequences.<br><br>How many reads were simulated in each simulation scenario? Did the authors vary the sequencing depth in the simulations?<br><br>We simulated all possible reads base by base with selected insert size in each scenario, and the number of reads in each scenario are not equal. The varied depth and the number of reads were listed for each scenario in updated Supplementary Table 6.<br><br><br>Minor<br>1. Section "Description in silico and real data": authors mentioned that simulated reads were selected from chr 18 of GRCh38 and then next sentence "Input fragments were selected from chr 18 in the GRCh37 assembly (hg19) of the human genome". Please, correct this if it's your typo, or clarify why different versions of reference genome were used for simulation.<br>All data used in the manuscript were based on simulation of chr1, although we have also simulated chr18. Thanks. We have revised the text.<br><br>2. Also they are still using the word "stimulated" instead of "simulated". And in the section "Availability of supporting data" the chromosome 1 was mentioned as chosen

Powered by Editorial Manager® and ProduXion Manager® from Aries Systems Corporation

	for simulation. Please correct it. Also typo on page 14-15: "We stimulated three kinds of reads, PE50, 90, and 50" should be 150.<br>As the answer to the above question, all descriptions in the manuscript were revised to chr1. Thank you, we have also revised the typo.<br><br>3. Legend to Figure 1: The sentence "Methylation sites were showed as read bases" do you mean "red" bases?<br>Thank you for your comments, we revised the typo.<br><br>4. The information for which virus was used for the simulation of the viral integration is mentioned only at the end of the manuscript (Availability of supporting data). Please provide this information in section "Description in silico and real data".<br>Thank you for your comments, we revised the text based on your suggestion.
**Additional Information:**	
**Question**	**Response**
Are you submitting this manuscript to a special series or article collection?	No
**Experimental design and statistics**<br><br>Full details of the experimental design and statistical methods used should be given in the Methods section, as detailed in our Minimum Standards Reporting Checklist. Information essential to interpreting the data presented should be made available in the figure legends.<br><br>Have you included all the information requested in your manuscript?	Yes
**Resources**<br><br>A description of all resources used, including antibodies, cell lines, animals and software tools, with enough information to allow them to be uniquely identified, should be included in the Methods section. Authors are strongly encouraged to cite Research Resource Identifiers (RRIDs) for antibodies, model organisms and tools, where possible.<br><br>Have you included the information requested as detailed in our Minimum Standards Reporting Checklist?	Yes
**Availability of data and materials**<br><br>All datasets and code on which the conclusions of the paper rely must be either included in your submission or deposited in publicly available repositories (where available and ethically appropriate), referencing such data using	Yes

a unique identifier in the references and in the “Availability of Data and Materials” section of your manuscript.<br><br>Have you have met the above requirement as detailed in our Minimum Standards Reporting Checklist?	

Manuscript
Click here to download Manuscript Manuscript_17Oct2017_v2.doc

BS-virus-finder: virus integration calling using bisulfite-sequencing data

Shengjie Gao[1,2,3,7,8,9*], Xuesong Hu[2,3*], Fengping Xu[3,10,13*], Changduo Gao[4], Kai Xiong[5], Xiao Zhao[2,11], Haixiao Chen[3,13], Shancen Zhao[3,7], Mengyao Wang[3], Dongke Fu[2], Xiaohui Zhao[6], Jie Bai[3], Likai Mao[3], Bo Li[2,3], Song Wu[8], Jian Wang[3], Shengbin Li[2,12], Huangming Yang[3, 7,11], Lars Bolund[9#], Christian N. S. Pedersen[1#]

[1] Bioinformatics Research Centre, Aarhus University, Aarhus, Denmark
[2] BGI-Forensic, Shenzhen 518083, China
[3] BGI-Shenzhen, Shenzhen 518083, China
[4] College of Computer Science & Technology, Qingdao University, Qingdao 266071, China
[5] Department of Veterinary Clinical and Animal Sciences, University of Copenhagen, Copenhagen, Denmark
[6] College of Mathematics & Statistics, Changsha University of Science & Technology, Changsha 410114, China
[7] James D. Watson Institute of Genome Sciences, Hangzhou 310058, China
[8] The Affiliated Luohu Hospital of Shenzhen University, Shenzhen University, Shenzhen 518000, China.
[9] Department of Biomedicine, Aarhus University, Aarhus, Denmark
[10] Department of Biology, University of Copenhagen, Copenhagen, Denmark
[11] BGI Education Center, University of Chinese Academy of Sciences.
[12] Shenzhen Key Laboratory of Forensics, BGI-Shenzhen, Shenzhen 518083, China.
[13] China National GeneBank, BGI-Shenzhen, Shenzhen 518120, China

[*] These authors contributed equally to this work
[#] These authors equally directed the work

Abstract

Background: DNA methylation plays a key role in regulation of gene expression and carcinogenesis. Bisulfite sequencing studies mainly focus on calling SNP, DMR, and ASM. Until now, only a few software tools focus on virus integration using bisulfite sequencing data.

Findings: We have developed a new and easy-to-use software tool, named as BS-virus-finder (BSVF, RRID: SCR_015727), to detect viral integration breakpoints in whole human genomes. The tool is hosted at https://github.com/BGI-SZ/BSVF.

Conclusions: BS-virus-finder demonstrates high sensitivity and specificity, and it is useful in epigenetic studies and to reveal the relationship between viral integration and DNA methylation. BS-virus-finder is the first software tool to detect virus by using bisulfite sequencing data.

Keyword: Virus integration, Bisulfite sequencing, Carcinogenesis

Findings

Introduction

DNA methylation plays crucial roles in many areas including development [1, 2] and X chromosome inactivation [3] by regulating genetic imprinting and epigenetic modification without altering DNA sequences. Previous studies have showed strong association of DNA methylation with cancer. The methylation status altering related carcinogenesis [4], cancer recurrence [5] and metastasis [6] has already been revealed by emerging bisulfite sequencing (BS) technology. BS technology can investigate DNA methylation changes with single-base accuracy. Treatment of DNA with bisulfite converts unmethylated cytosine residues to uracil, but leaves 5-methylcytosine residues unmodified [7]. Thus, bisulfite treatment introduces specific changes in the DNA sequence that depend on the methylation status of individual cytosine residues, yielding single-nucleotide resolution information about the methylation status of a segment of DNA (Figure 1). Various analyses can be performed on the altered sequences to retrieve this information. BS technology can reveal differences between cytosines and thymidine and sequence change resulting from bisulfite conversion. For the bases without methylation, all C will change to T on both strands. After directional library preparation, we have two different conversions: The Watson, and the Crick strand, as shown in Figure 1. On the Watson strand, methylated C remains C, and unmethylated C changes to T. On the Crick strand, the reverse complement happens, i.e. methylated C remains C but in sequenced reads it is reverse complemented to G, and unmethylated C changes to T, leading to

the reverse complement base A in sequenced reads. Since base C can either be methylated or unmethylated, we can use IUPAC nucleotide code “Y” and “R” to represent C/T and G/A respectively. So, after bisulfite treatment, base C changes to Y on the Watson strand, and base G changes to R on the Crick strand.

Whole-genome based bisulfite sequencing (WGBS) has been developed to detect DNA methylation. Recent clinical studies showed that DNA methylation is associated with viral integration [8, 9]. Whole-genome BS (WGBS) data can be analyzed to investigate the sequence mapping and alignment via BSMAP [10], Bismark [11] and bwa-meth [12], to detect DMR (different methylation regions) via software QDMR [13], DMAP [14] and SMAP [15], to identify SNP (single sucleotide polymorphism) via software BS-SNPer [16] and Bis-SNP [17], to find ASM (allele-specific DNA methylation) via SMAP [15], Methy-Pipe [18]. However, none of them can be used for virus integration loci calling, and no software tool is currently available to detect virus integration loci by analyzing BS data. Therefore, we have developed a software tool to detect the virus integration loci by genome-wide BS analysis.

Description in silico and real data.

Different types of PE (paired-end) reads (50bp, 90bp, 150bp) that include 700 breakpoints in chromosome 1 (chr 1) of GRCh38 were simulated in our study. Input fragments of 50 to 400 bp were randomly selected from chr 1 in the GRCh38 assembly of the human genome. HBV genome (GenBank: X04615.1) was used in our simulation. Its integration length was between 45 bp and 180 bp. We cut HBV containing segments with given pair-end insert size at all

possible positions on every integration events. After alignment, mapping accuracy of each of the 17 different types of reads mapping was calculated (Figure 2). Mapping accuracy varied among the 17 types of read mappings in our simulation (Figure S1, S2, S3). In summary, the accuracies of several kinds of the reads mappings were low (Table S1, S2, S3), which may raise false-negative rate. Generally, however, bwa-meth [12] performed very well.

Bisulfite sequencing is a marvelous and sophisticated technique to study DNA cytosine methylation. Bisulfite treatment followed by PCR amplification specifically converts unmethylated cytosine to thymine. By cooperating with next generation sequencing technology, it is able to detect the methylation status of every cytosine in the whole genome. Moreover, longer reads make it possible to achieve higher accuracy. Besides simulated data, the PLC/PRF/5 hepatocellular carcinoma cell lines were from American Type Culture Collection (ATCC, Manassas, VA) were cultured as previously described [19]. The cell line was validated by STR makers (Figure S4). We performed WGS and WGBS sequencing of this cell line, the result is showed in Table S4. Table 1 shows the analysis result for WGS data, which was compared with the output results analyzed by Vy-per [20], virus-clip[21] and Virus Finder2 [22].

Methods

Sample preparation

PLC/PRF/5 hepatocellular carcinoma cell line was obtained from American Type Culture Collection (ATCC, Manassas, VA) and were cultured as previously described [19] , which was

also validated by STR makers (Figure S4). Then totally 15 μg DNA was extracted to perform WGS and WGBS sequencing. Sample concentration was detected by fluorometer (QubitFluorometer, Invitrogen). Sample integrity and purification was determined by Agarose Gel Electrophoresis.

Whole genome sequencing

About 1.5 μg gDNA was sonicated to 100-300 bp fragment genome DNA by Sonication (Covaris), purified with QIAquick PCR Purification Kit (Qiagen). Adapter ligation and target insert size fragements recovering, and quantifying library by real-time quantitative PCR (QPCR) (TaqMan Probe) was then performed. The qualified library was sequenced on an Illumina Hiseq X Ten platform and 150bp paired-end reads were obtained. Totally, around 90 G clean data were generated.

Whole genome bisulfite sequencing

About 3 μg gDNA were sonicated to 100-300 bp by Sonication (Covaris), purified with MiniElute PCR Purification Kit (QIAGEN). A single 'A' nucleotide was added to the 3' ends of the blunt fragments. Methylated adapters were then purified and added to the 5' and 3' ends of each strand in the genomic fragment. Sizes 300-400bp were selected. DNA was then purified with QIAquick Gel Extraction kit (QIAGEN) and bisulfite treated with Methylation-Gold kit (ZYMO). Finally PCR was conducted and sizes 350-400bp were selected and purified with QIAquick Gel Extraction kit (QIAGEN). Qualified library was amplified on cBot to generate the cluster on the flowcells (TruSeq PE Cluster Kit

V3–cBot–HS, Illumina). The flowcells were sequenced for 150 bp pair end reads on HiSeq X Ten platform and more than 90G clean data were generated.

Data analysis

The reads coverage situation for one integration is shown in Figure 3. Four steps were implemented to detect virus integration:

1. Alignment

We use bwa-meth [12] to align bisulfite treated sequencing reads to a hybrid reference that contains both human genome and virus sequences. For chimeric reads from the junction parts, BWA-MEM [23] will align it to one organism and mark the unmapped part as soft clipping, which is in fact from the other organism. This enables us to find breakpoints directly from the alignment.

2. Clustering

After alignment, the result was filtered. We select read pairs with one read match by the following criterion: the Phred-scaled mapping quality is bigger than 30 (>=30), and at least one soft clipping is longer than 5 bp (>=5). The mapped parts of reads, which is marked as “M” by its CIGAR string, cover the human reference genome. For paired reads, we also add the gap between two mapped reads to their covered region, making read 1 and read 2 be continuous covered on human reference. Each continuous region with at least 1 bp overlap are defined as a cluster. All reads involved are selected to form the cluster. The remaining soft clippings are viral junction candidates. Read pairs with one read mapped on virus also indicate potential virus junction between the read pair.

3. Assembling

Within one cluster, all soft clipping start sites are collected. The position with the most abundance of start sites is identified as the most likely candidate breakpoint. All clipping sequences in the cluster are extracted and aligned together. A restore algorithm was used to calculate the most possible base in each position based on the aligned bases and its sequencing quality. The algorithm is based on a Bayesian model, where we compute the posteriori probability estimation for A, C, G, T as:

$$\begin{aligned} P(T_i \mid D) &= \frac{P(T_{Wi})P(D \mid T_{Wi})}{\sum_{x=1}^{S} P(T_{Wx})P(D \mid T_{Wx})} \times \frac{P(T_{Ci})P(D \mid T_{Ci})}{\sum_{x=1}^{S} P(T_{Cx})P(D \mid T_{Cx})} \\ &= C_0 \times P(D \mid T_{Wi}) \times P(D \mid T_{Ci}) \\ C_0 &= \frac{P(T_{Wi})}{\sum_{x=1}^{S} P(T_{Wx})P(D \mid T_{Wx})} \times \frac{P(T_{Ci})}{\sum_{x=1}^{S} P(T_{Cx})P(D \mid T_{Cx})} \end{aligned} \quad (1)$$

.

Here, D is the observation of the NGS reads on given position. P(Ti|D) is the likelihood component, which can be interpreted as the probability of observing D when the true genotype is T_i. D_W be a realization (or observation) of the NGS reads in the Watson strand. D_C be a realization (or observation) of the NGS reads in Crick strand. $P(T_{Wi}|D)$ is the likelihood component, which can be interpreted as the probability of observing D when the true genotype is T_{Wi}. $P(T_{Ci}|D)$ is the likelihood component, which can be interpreted as the probability of observing D when the true genotype is T_{Ci}. At each virus location, prior probability P(Ti) of each genotype Ti was set according to the Table S5. The likelihood P(D|Ti) for the assumed genotype Ti was calculated from the observed allele types in the sequencing reads in formula 2. Thus, on the Watson strand it is $P(D_W|T_i)$, on the Crick strand it is $P(D_C|T_i)$. We defined the likelihood of observing allele d_k in a read for a possible haploid genotype T as $P(d_k|T)$, and on

the Watson strand it is $P(d_{Wk}|T)$, and on the Crick strand it is $P(d_{Ck}|T)$. So, for a set of n observed alleles at a locus, $D = \{d_1, d_2, \ldots, d_n\}$ on each strand, these probabilities are computed as shown by formula 3 & 4, where Q stands for the base quality from the fastaq file.

$$P(D_W \mid T_i) = \prod_{k=1}^{m} P(d_{Wk} \mid T), P(D_C \mid T_i) = \prod_{k=1}^{n} P(d_{Ck} \mid T). \quad (2)$$

$$P(d_{Wk} \mid T) = \begin{cases} 1 - 10^{-\frac{Q}{10}} & (T \in \{A, C, G\}) \\ \frac{1 - 10^{-\frac{Q}{10}}}{2} & (T \in \{T\}) \end{cases}, \quad (3)$$

$$P(d_{Ck} \mid T) = \begin{cases} 1 - 10^{-\frac{Q}{10}} & (T \in \{C, G, T\}) \\ \frac{1 - 10^{-\frac{Q}{10}}}{2} & (T \in \{A\}) \end{cases}. \quad (4)$$

We used “Y” and “R” to represent C/T and G/A respectively (IUPAC nucleotide code). If a region is covered by both the Watson strand and the Crick strand, we were able to deduce the original base from Y or R by calculation.

4. Detection of viral integrations

The assembled clipping regions above were mapped to the given virus reference sequence with a Smith-Waterman local alignment tool from EMBOSS package [24], which support IUPAC DNA codes Y and R. Virus fragment location is extracted from the alignment results.

Discussion

In summary, we have implemented the first software tool to detect virus integration using BS data. Our software is based on bwa-meth, and by assembling and aligning soft-clip regions, it can find the virus breakpoints. However, accuracy of reads surrounding the breakpoints needs to be further improved. Virus usually integrates into regions that are homologous to both human and virus (micro-homologous) [25]. Therefore, we consider the breakpoints predicted by our software tool that are within 10 bp of a real breakpoint as being correctly identified

(Figure S2). With this definition, the accuracy of our predicted breakpoints can reach over 70%. Our results will be useful for analyzing BS data and related applications. Some of the results come with only location on human genome, and has the virus location missing. This may be due to the shortage of virus fragments. We simulated three kinds of reads, PE50, 90, and 150 with various lengths, and further simulated virus-inserted fragment with different length as well (Table S6), thus all cases described in Figure 2 are mimicked here. All simulation sampled all possible reads, base by base with fixed insert size. As the result in Table S6 showed, the longer the reads, the more accurate the prediction can be achieved. In particular, for read lengths around 50 bp, BS-virus-finder is capable to find the virus integration with an accuracy of more than 70%; for the read lengths between 90bp and 150bp, BS-virus-finder is capable to find the virus integration with an accuracy of more than 90%. Besides simulated data, we have performed WGS and WGBS sequencing of the PLC/PRF/5 hepatocellular carcinoma cell line (Table S4). As the results showed, when the length of input is larger than 150bp, the analysis result of WGBS is similar to the one of WGS. Additionally, BS-virus-finder is able to find breakpoints in 8 out of 9 regions which are identified by FISH [8]. Based on these experimental results, we believe that BS-virus-finder is a powerful software tool to analyze virus-integration using BS data.

Availability and requirements

Project Name: BS-virus-finder: virus integration calling using bisulfite-sequencing data

Project home page: https://github.com/BGI-SZ/BSVF [26]

Operating system: Linux

Programming language: Perl, Python, C

License: LGPL v3

Availability of supporting data

Data used in this paper is simulated based on random insertion of HBV sequence to human chromosome 1 sequence. A Perl script named “simVirusInserts.pl” is included, and our simulation schema is coded within. We have run the simulation several times and the result shows no significant difference. The PLC/PRF/5 hepatocellular carcinoma cell lines were from American Type Culture Collection (ATCC, Manassas, VA) and sequenced by HiSeq X Ten System from Novogene company. WGS and WGBA data have been submitted to NCBI SRA project PRJNA400455.

Competing interests

The authors declare that they have no competing interests.

Authors' contributions

CP, LB and HY conceptualized the project. SG, XH, SL and JW designed BSVF and developed its accompanying utilities. SG, XH, CG, XZ, MW and SZ developed the protocol. FX, DF, HC and JB conducted experiment. SG, XH, BL and SW undertook the analysis. KX, LM, SG, XH, LB and CP wrote and approved the final version of the manuscript. All authors read and approved the final manuscript.

Acknowledgements

We appreciate the supporting of Xiaolin Liang and Hengtong Li in College of Mathematics &

Statistics, Changsha University of Science & Technology, for their contributing advice to our research. This work was funded by the National Natural Science Foundation of China (81602477) and Shenzhen Municipal Government of China (ZDSYS201507301424148).

References

1. Wang Y, Shang Y: Epigenetic control of epithelial-to-mesenchymal transition and cancer metastasis. Experimental cell research 2013, 319(2):160-169.
2. O'Doherty AM, Magee DA, O'Shea LC, Forde N, Beltman ME, Mamo S, Fair T: DNA methylation dynamics at imprinted genes during bovine pre-implantation embryo development. BMC developmental biology 2015, 15:13.
3. Cotton AM, Price EM, Jones MJ, Balaton BP, Kobor MS, Brown CJ: Landscape of DNA methylation on the X chromosome reflects CpG density, functional chromatin state and X-chromosome inactivation. Human molecular genetics 2015, 24(6):1528-1539.
4. Kamdar SN, Ho LT, Kron KJ, Isserlin R, van der Kwast T, Zlotta AR, Fleshner NE, Bader G, Bapat B: Dynamic interplay between locus-specific DNA methylation and hydroxymethylation regulates distinct biological pathways in prostate carcinogenesis. Clinical epigenetics 2016, 8:32.
5. Haldrup C, Mundbjerg K, Vestergaard EM, Lamy P, Wild P, Schulz WA, Arsov C, Visakorpi T, Borre M, Hoyer S et al: DNA methylation signatures for prediction of biochemical recurrence after radical prostatectomy of clinically localized prostate cancer. Journal of clinical oncology : official journal of the American Society of Clinical Oncology 2013, 31(26):3250-3258.
6. Kim JH, Dhanasekaran SM, Prensner JR, Cao X, Robinson D, Kalyana-Sundaram S, Huang C, Shankar S, Jing X, Iyer M et al: Deep sequencing reveals distinct patterns of DNA methylation in prostate cancer. Genome research 2011, 21(7):1028-1041.
7. Darst RP, Pardo CE, Ai L, Brown KD, Kladde MP: Bisulfite sequencing of DNA. Current protocols in molecular biology / edited by Frederick M Ausubel [et al] 2010, Chapter 7:Unit 7 9 1-17.
8. Watanabe Y, Yamamoto H, Oikawa R, Toyota M, Yamamoto M, Kokudo N, Tanaka S, Arii S, Yotsuyanagi H, Koike K et al: DNA methylation at hepatitis B viral integrants is associated with methylation at flanking human genomic sequences. Genome research 2015, 25(3):328-337.
9. Lillsunde Larsson G, Helenius G, Sorbe B, Karlsson MG: Viral load, integration and methylation of E2BS3 and 4 in human papilloma virus (HPV) 16-positive vaginal and vulvar carcinomas. PloS one 2014, 9(11):e112839.
10. Xi Y, Li W: BSMAP: whole genome bisulfite sequence MAPping program. BMC bioinformatics 2009, 10:232.
11. Krueger F, Andrews SR: Bismark: a flexible aligner and methylation caller for

Bisulfite-Seq applications. Bioinformatics 2011, 27(11):1571-1572.
12. Pedersen BS EK, De S, Yang IV, Schwartz DA: Fast and accurate alignment of long bisulfite-seq reads. eprint arXiv 2014.
13. Zhang Y, Liu H, Lv J, Xiao X, Zhu J, Liu X, Su J, Li X, Wu Q, Wang F et al: QDMR: a quantitative method for identification of differentially methylated regions by entropy. Nucleic acids research 2011, 39(9):e58.
14. Stockwell PA, Chatterjee A, Rodger EJ, Morison IM: DMAP: differential methylation analysis package for RRBS and WGBS data. Bioinformatics 2014.
15. Gao S, Zou D, Mao L, Zhou Q, Jia W, Huang Y, Zhao S, Chen G, Wu S, Li D et al: SMAP: a streamlined methylation analysis pipeline for bisulfite sequencing. GigaScience 2015, 4:29.
16. Gao S, Zou D, Mao L, Liu H, Song P, Chen Y, Zhao S, Gao C, Li X, Gao Z et al: BS-SNPer: SNP calling in bisulfite-seq data. Bioinformatics 2015, 31(24):4006-4008.
17. Liu Y, Siegmund KD, Laird PW, Berman BP: Bis-SNP: Combined DNA methylation and SNP calling for Bisulfite-seq data. Genome biology 2012, 13(7):R61.
18. Jiang P, Sun K, Lun FM, Guo AM, Wang H, Chan KC, Chiu RW, Lo YM, Sun H: Methy-Pipe: an integrated bioinformatics pipeline for whole genome bisulfite sequencing data analysis. PloS one 2014, 9(6):e100360.
19. Carr BI, Cavallini A, Lippolis C, D'Alessandro R, Messa C, Refolo MG, Tafaro A: Fluoro-Sorafenib (Regorafenib) effects on hepatoma cells: growth inhibition, quiescence, and recovery. J Cell Physiol 2013, 228(2):292-297.
20. Forster M, Szymczak S, Ellinghaus D, Hemmrich G, Ruhlemann M, Kraemer L, Mucha S, Wienbrandt L, Stanulla M, Group UFOSCwI-BS et al: Vy-PER: eliminating false positive detection of virus integration events in next generation sequencing data. Sci Rep 2015, 5:11534.
21. Ho DW, Sze KM, Ng IO: Virus-Clip: a fast and memory-efficient viral integration site detection tool at single-base resolution with annotation capability. Oncotarget 2015, 6(25):20959-20963.
22. Wang Q, Jia P, Zhao Z: VERSE: a novel approach to detect virus integration in host genomes through reference genome customization. Genome Med 2015, 7(1):2.
23. Li H: Aligning sequence reads, clone sequences and assembly contigs with BWA-MEM. eprint arXiv 2013:3.
24. Rice P, Longden I, Bleasby A: EMBOSS: the European Molecular Biology Open Software Suite. Trends in genetics : TIG 2000, 16(6):276-277.
25. Hu Z, Zhu D, Wang W, Li W, Jia W, Zeng X, Ding W, Yu L, Wang X, Wang L et al: Genome-wide profiling of HPV integration in cervical cancer identifies clustered genomic hot spots and a potential microhomology-mediated integration mechanism. Nature genetics 2015, 47(2):158-163.
26. Bisulfite Sequencing Virus integration Finder. https://github.com/BGI-SZ/BSVF. Accessed 16 Oct 2017.

Table 1. The comparison of BS-virus-finder with other software using real data.

Chr	BSVF			Vy-per			virus-clip			Virus Finder2		
	HB	VB	VE	HB	VB	VE	HB	VB	VE	HB	VB	VE
chr1	143272758	2945	3102									
chr2	-			-			52018758	207	281			
chr3*	131451702	1212	1322	-			131451701	1282	1403	131451701	1405	
chr3*	131453124	1416	1515	-			131453353	1416	1538			
chr4*	180586417	136	378							180586416	59	
chr4*	180587608	394	594	180586607	167	231	180587608	500	632	180587607	634	
chr5*	1297478	1174	1315	-			1297478	1241	1385	1297477	1388	
chr7	110894616	2739	2748									
chr8*	35446380	2389	2459	35446214	2402	2455	35446601	2390	2519	35446392	2396	2608
chr8	-						106944290	698	1077			
chr11*	65040943	2631	2767	-			-			65040964	2532	
chr12*	109573899	721	815	109573677	668	734	109573899	705	815			
chr13	33088123	1521	1603	-			-					
chr13	33088561	1917	2066	-			33088561	1995	2133	33088560	2133	
chr16*	69947046	2055	2826									
chr16*	70169959	2055	2735							70169971	2064	2240
chr16	74425602	2062	2665									
chr17*	82105786	407	489	82105984	368	435	82105783	347	489			
chr17*	82107626	2177	2321	-			82107710	2048	2159	82107625	2045	
chr19	41783064	687	804	-			41782971	761	905			
chr20	20473566	2415	2565									

BSVF used WGBS data, and other software used WGS data.

* supported by previous FISH experiments [8].

HB: Host breakpoint.

VB: Virus Begin is the revealed left most position on virus.

VE: Virus End is the right most position on virus.

Figure 1. The illustration of bisulfite-altered sequence to the original.

Figure 2. Principal types of mapping reads around the viral integration site.

Figure 3. A demo plot of one viral integration cluster in its pre-insertion form.

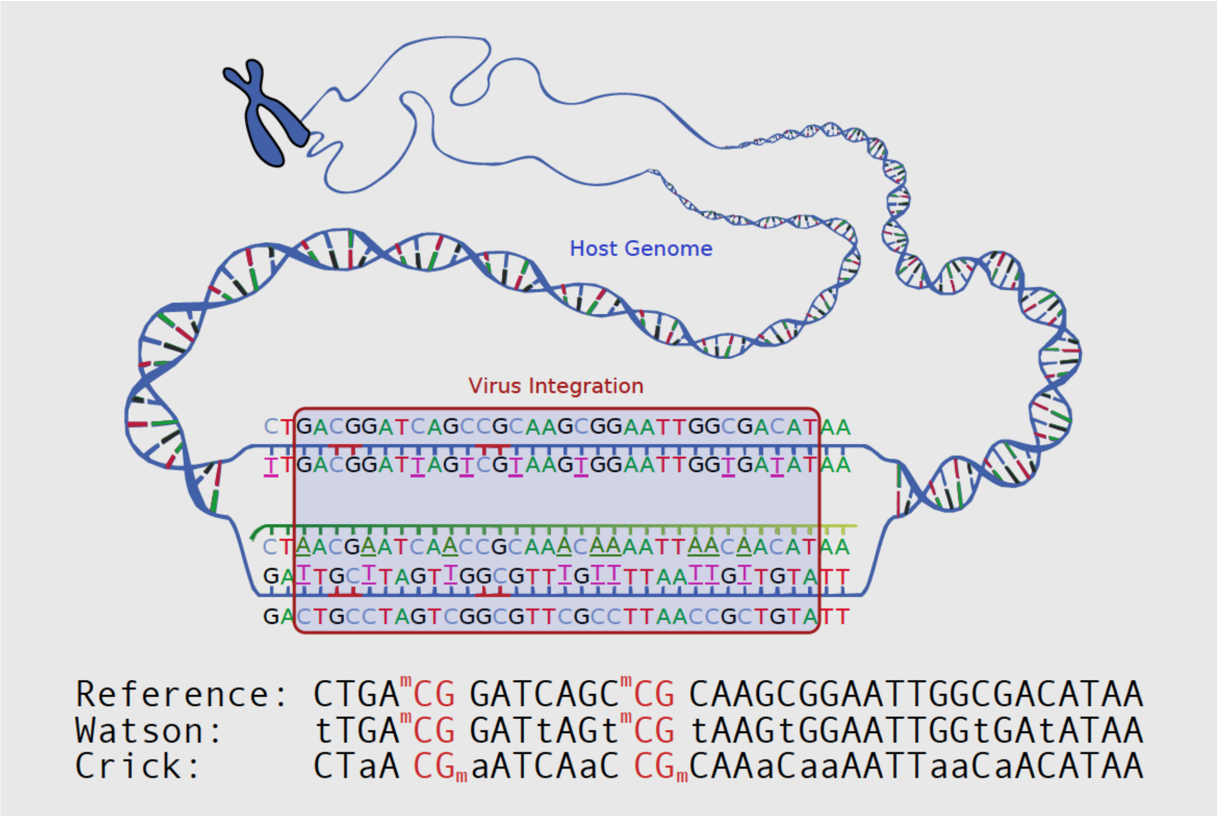

Figure 1. The illustration of bisulfite-altered sequence to the original.

Reference is the original sequence prior to bisulfite treatment. After directional library preparation, we have two different conversion: Watson and Crick strand.

Methylation sites were showed as red bases. Bisulfite treated base may alter the original base from C to T. m indicated methylation-modified base. Low-case letter indicates the bisulfite-altered base.

As we can see, half of the probability of each T is C in Watson strand and half of the probability of each A is G in Crick strand.

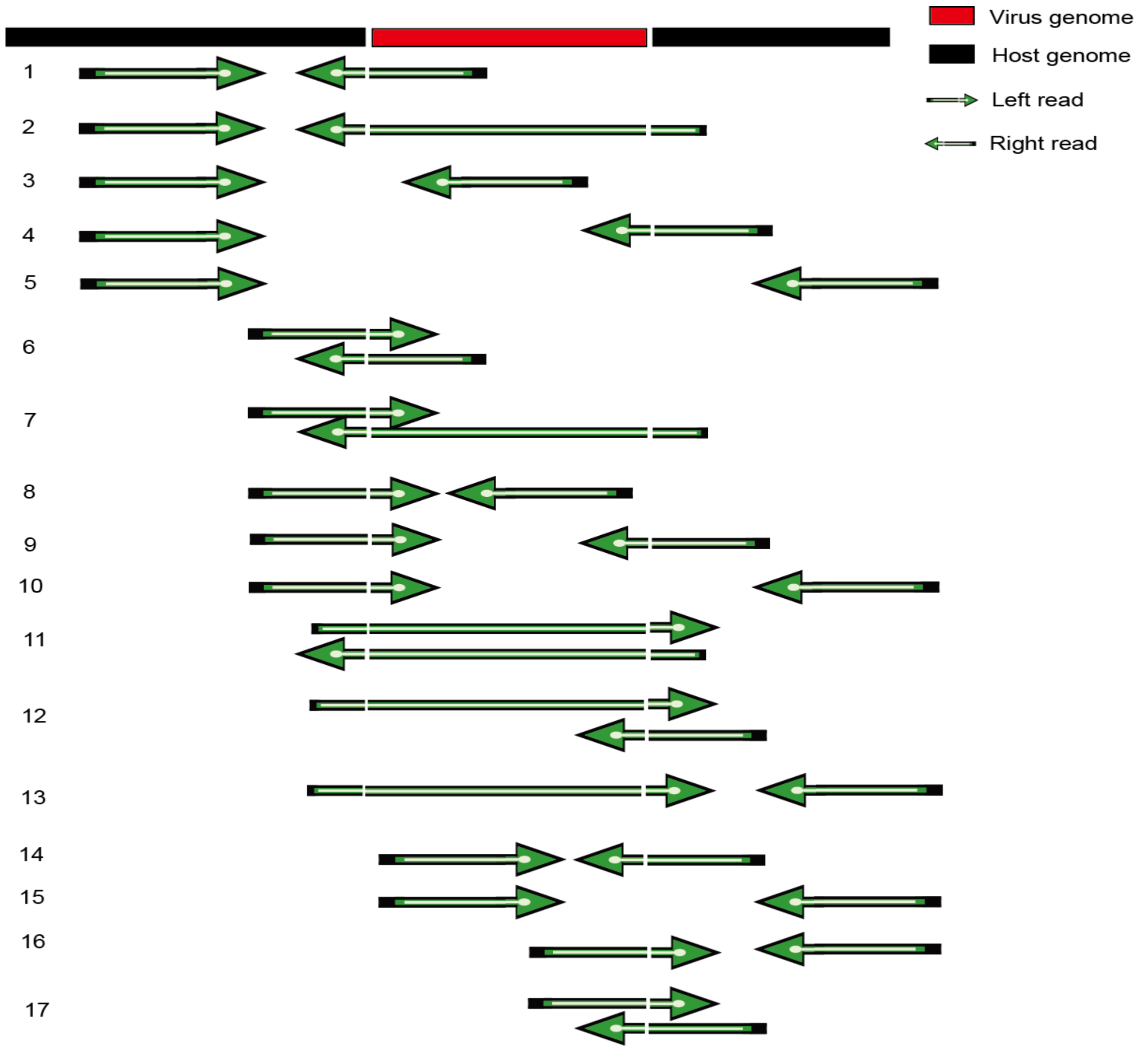

Figure 2. Principal types of mapping reads around the viral integration site. Red bar, the virus sequence inserted in host genome; Green arrow, mapping reads with different directions; Breakpoints indicate logical division between host genome and virus, which are physically linked.

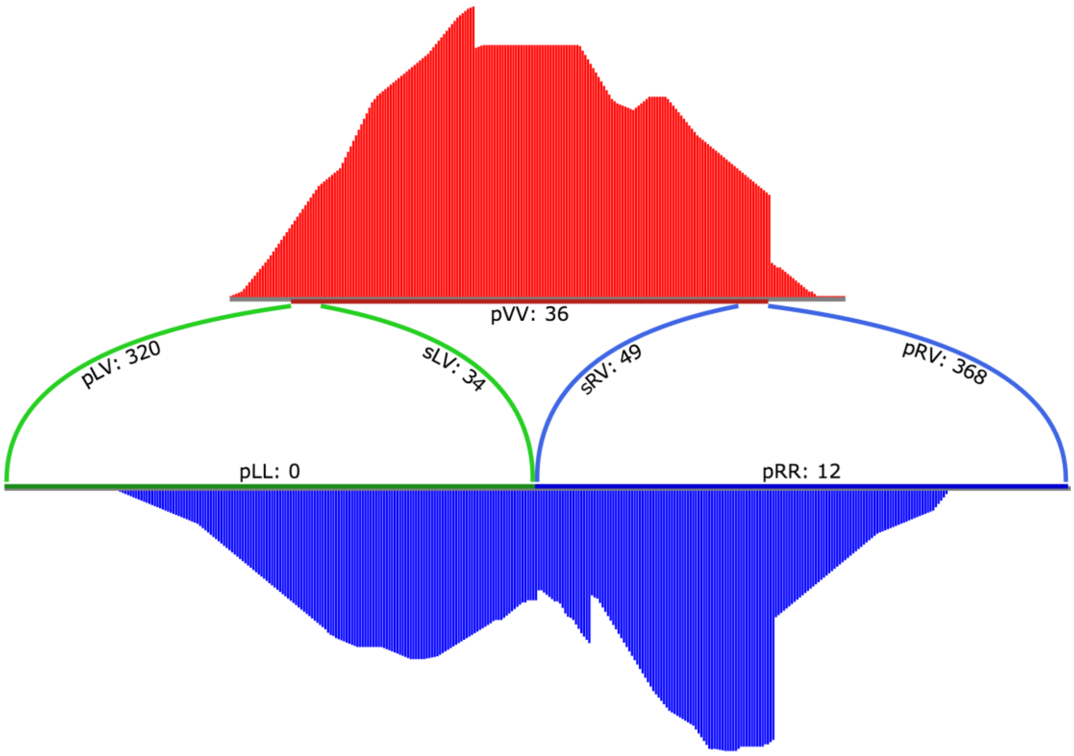

Figure 3. A demo plot of one viral integration cluster in its pre-insertion form.

Plot was randomly selected from simulated breakpoints.

The red virus fragment(V) above will be inserted into the center point of the green(L) and blue(R) human fragment below to form the sequenced sample. Bars show the coverage depth of sequencing reads.

Curves represent the linkage events supported by pair-end sequencing reads, and the number besides shows the read count.

Click here to access/download
Supplementary Material
Supplementary-bsfinder.docx

Personal Cover
Click here to download Personal Cover CoverLetter-17Oct2017.pdf

AARHUS UNIVERSITY
BIOINFORMATICS RESEARCH CENTRE (BIRC)

GigaScience Editorial Office

Submission of final version of paper

Dear Editor in Chief

Thank you very much for accepting our paper "BS-virus-finder: virus integration calling using bisulfite-sequencing data" (GIGA-D-17-00032R1) cf. your e-mail dated September 29, 2017. We have addressed the points raised by the reviewers to the best of our abilities, and as outlined in our response to reviewers. We hope that you find everything in order.

Kind regards

Christian Nørgaard Storm Pedersen
(on behalf of all the authors of the paper)

Bioinformatics Research Centre (BiRC)

Christian Nørgaard Storm Pedersen

Centre director, Associate professor

Date: 17 October 2017

Direct Tel.: +45 87155559
Mobile Tel.: +45 27782810
E-mail: cstorm@birc.au.dk

Web: au.dk/en/cstorm@birc

Sender's CVR no.: 31119103

Bioinformatics Research Centre
Aarhus University
C.F.Møllers Allé 8
DK-8000 Aarhus C
Denmark

Tel.: +45 87155557
Fax: +45 87154102
E-mail: admin@birc.au.dk
http://birc.au.dk/
