# Supplementary material for: BS-virus-finder: virus integration calling using bisulfite sequencing data
Source: Gigascience. 2017 Dec 18;7(1):1–7. doi: 10.1093/gigascience/gix123 (PMC5788064; doi:10.1093/gigascience/gix123)
Supplement: Reviewer_1_Revision_1_(Attachment).pdf [file gix123_reviewer_1_revision_1_(attachment).pdf]

Re-review on manuscript

BS-virus-finder: virus integration calling using bisulfite-sequencing data

Shengjie Gao, Ph.D; Xuesong Hu; Changduo Gao; Kai Xiong; Fengping Xu; Xiao Zhao; Haixiao Chen; Shancen Zhao; Mengyao Wang; Dongke Fu; Xiaohui Zhao; Jie Bai; Bo Li; Song Wu; Shengbin Li; Huanming Yang; Lars Bolund; Christian Pedersen.

by Lada A Koneva, PhD

Introduction

The reviewed manuscript (BS-virus-finder: virus integration calling using bisulfite-sequencing data) describes developed software to detect viral integration breakpoints in whole-genome bisulfite-sequencing data (WGBS).

The manuscript was improved according to my suggested concerns and questions, but there are still some questions.

Questions

As a consequence of choosing HBV for simulation and PLC/PRF/5 hepatocellular carcinoma cell lines (which harboring HBV) as a real data, there is still unanswered my question about how this algorithm will work in case of HPV integration. There are about 170 types of HPV, with high commonality between different types, and about 15 types are carcinogenic. How authors suggest creating a “hybrid reference that contains both human genome and virus sequences” in case of HPV? Which viral types could be chosen for alignment of assembled clipping regions in case of HPV contamination? Probably this algorithm could not accurately predict which type(s) of HPV contaminates the sample. It would be useful if authors could provide their thoughts if this algorithm also will work in case of virus like HPV or this should be restricted to analysis of HBV integration only.

How many reads were simulated in each simulation scenario? Did the authors vary the sequencing depth in the simulations?

Minor

1. Section “Description in silico and real data”: authors mentioned that simulated reads were selected from chr 18 of GRCh38 and then next sentence “Input fragments were selected from chr 18 in the GRCh37 assembly (hg19) of the human genome”. Please, correct this if it’s your typo, or clarify why different versions of reference genome were used for simulation.
2. Also they are still using the word “stimulated” instead of “simulated”. And in the section “Availability of supporting data” the chromosome 1 was mentioned as chosen for simulation. Please correct it. Also typo on page 14-15: “We stimulated three kinds of reads, PE50, 90, and 50” should be 150.

3. Legend to Figure 1: The sentence “Methylation sites were showed as read bases” do you mean “red” bases?
4. The information for which virus was used for the simulation of the viral integration is mentioned only at the end of the manuscript (Availability of supporting data). Please provide this information in section “Description in silico and real data”.
