# Supplementary material for: BS-virus-finder: virus integration calling using bisulfite sequencing data
Source: Gigascience. 2017 Dec 18;7(1):1–7. doi: 10.1093/gigascience/gix123 (PMC5788064; doi:10.1093/gigascience/gix123)
Supplement: Supplemental material [file gix123_supp.docx]

## Table S1. Alignment accuracy rate around the breakpoint region using PE50 data.

| **Type** | **# of right match** | **# of error match** | **Rate of mapping correctly** |
| --- | --- | --- | --- |
| 1 | 656482 | 56747 | 0.92 |
| 2 | 353425 | 38513 | 0.90 |
| 3 | 68142 | 14487 | 0.82 |
| 4 | 260049 | 21269 | 0.92 |
| 5 | 280897 | 30463 | 0.90 |
| 6 | 44775 | 37930 | 0.54 |
| 7 | 41521 | 31821 | 0.57 |
| 8 | 12513 | 4916 | 0.72 |
| 9 | 57399 | 16161 | 0.78 |
| 10 | 259849 | 21462 | 0.92 |
| 11 | 1131 | 14991 | 0.07 |
| 12 | 41703 | 31217 | 0.57 |
| 13 | 352703 | 39231 | 0.90 |
| 14 | 12306 | 5196 | 0.70 |
| 15 | 68370 | 13984 | 0.83 |
| 16 | 655902 | 57389 | 0.92 |
| 17 | 45393 | 37352 | 0.55 |

## Table S2. Alignment accuracy rate around the breakpoint region using PE90 data.

| **Type** | **# of right match** | **# of error match** | **Rate of mapping correctly** |
| --- | --- | --- | --- |
| 1 | 1153852 | 20882 | 0.98 |
| 2 | 752984 | 33797 | 0.96 |
| 3 | 98128 | 3581 | 0.96 |
| 4 | 361285 | 8617 | 0.98 |
| 5 | 409011 | 5757 | 0.99 |
| 6 | 129463 | 8017 | 0.94 |
| 7 | 83495 | 3146 | 0.96 |
| 8 | 53760 | 1820 | 0.97 |
| 9 | 143581 | 4011 | 0.97 |
| 10 | 361775 | 7962 | 0.98 |
| 11 | 1418 | 184 | 0.89 |
| 12 | 83254 | 3362 | 0.96 |
| 13 | 752332 | 34515 | 0.96 |
| 14 | 53497 | 2002 | 0.96 |
| 15 | 97969 | 3779 | 0.96 |
| 16 | 1152494 | 22803 | 0.98 |
| 17 | 128593 | 8903 | 0.94 |

## Table S3. Alignment accuracy rate around the breakpoint region using PE150 data.

| **Type** | **# of right match** | **# of error match** | **Rate of mapping correctly** |
| --- | --- | --- | --- |
| 1 | 1655410 | 12564 | 0.99 |
| 2 | 1242514 | 16742 | 0.99 |
| 3 | 209482 | 1760 | 0.99 |
| 4 | 562992 | 5010 | 0.99 |
| 5 | 411997 | 4783 | 0.99 |
| 6 | 326876 | 2615 | 0.99 |
| 7 | 96330 | 1058 | 0.99 |
| 8 | 87250 | 491 | 0.99 |
| 9 | 250960 | 1796 | 0.99 |
| 10 | 563037 | 4626 | 0.99 |
| 11 | 2939 | 210 | 0.93 |
| 12 | 96368 | 1139 | 0.99 |
| 13 | 1243970 | 15269 | 0.99 |
| 14 | 87202 | 584 | 0.99 |
| 15 | 209309 | 1933 | 0.99 |
| 16 | 1656559 | 12012 | 0.99 |
| 17 | 326806 | 3247 | 0.99 |

## Table S4. Mapping statistics of cell line sequencing data

|  | WGS | WGBS |
| --- | --- | --- |
| Average sequencing depth | 39.75 | 47.65 |
| Coverage | 94.04% | 94.22% |
| Coverage ≥4X | 93.70% | 93.75% |
| Coverage ≥10X | 93.15% | 92.37% |
| Coverage ≥20X | 89.89% | 84.19% |

## Table S5. The prior probability of bayesian model used in resoring process for bisulfite sequencing of integrated virus.

|  | P-Watson | P-Crick |
| --- | --- | --- |
| A | 0.25 | 0.125 |
| T | 0.125 | 0.25 |
| C | 0.25 | 0.25 |
| G | 0.25 | 0.25 |

## Table S6. The performance of BS-virus-finder *in silico* with different read length and insert sizes.

| **Read Length** | **PE Insert Length** | **Virus Length** | **True Positive** | | **HumOnly** | | **VirNA** | | **HumCalled %** | **False Positive** | | **Fraqment Len** | **PE Cnt** | **Bases** | **Depth** |
| --- | --- | --- | --- | --- | --- | --- | --- | --- | --- | --- | --- | --- | --- | --- | --- |
|  |  |  | **Cnt** | **%** | **Cnt** | **%** | **Cnt** | **%** |  | **Cnt** | **%** |  |  |  |  |
| **50** | 60 | 25 | 454 | 64.86% | 22 | 3.14% | 119 | 17.00% | 85.00% | 1 | 0.14% | 145 | 86 | 8600 | 59.31 |
|  | 80 | 25 | 438 | 62.57% | 30 | 4.29% | 174 | 24.86% | 91.71% | 7 | 1.00% | 185 | 106 | 10600 | 57.30 |
|  | 80 | 67 | 640 | 91.43% | 2 | 0.29% | 0 | 0.00% | 91.71% | 4 | 0.57% | 227 | 148 | 14800 | 65.20 |
|  | 120 | 5 | 51 | 7.29% | 479 | 68.43% | 52 | 7.43% | 83.14% | 1 | 0.14% | 245 | 126 | 12600 | 51.43 |
|  | 120 | 10 | 76 | 10.86% | 428 | 61.14% | 160 | 22.86% | 94.86% | 9 | 1.29% | 250 | 131 | 13100 | 52.40 |
|  | 120 | 20 | 512 | 73.14% | 29 | 4.14% | 131 | 18.71% | 96.00% | 1 | 0.14% | 260 | 141 | 14100 | 54.23 |
|  | 120 | 25 | 450 | 64.29% | 28 | 4.00% | 191 | 27.29% | 95.57% | 3 | 0.43% | 265 | 146 | 14600 | 55.09 |
|  | 120 | 67 | 581 | 83.00% | 13 | 1.86% | 74 | 10.57% | 95.43% | 3 | 0.43% | 307 | 188 | 18800 | 61.24 |
|  | 120 | 100 | 673 | 96.14% | 1 | 0.14% | 0 | 0.00% | 96.29% | 11 | 1.57% | 340 | 221 | 22100 | 65.00 |
|  | 250 | 67 | 262 | 37.43% | 266 | 38.00% | 20 | 2.86% | 78.29% | 0 | 0.00% | 567 | 318 | 31800 | 56.08 |
| **90** | 100 | 45 | 569 | 81.29% | 0 | 0.00% | 109 | 15.57% | 96.86% | 60 | 8.57% | 245 | 146 | 26280 | 107.27 |
|  | 150 | 45 | 570 | 81.43% | 2 | 0.29% | 115 | 16.43% | 98.14% | 38 | 5.43% | 345 | 196 | 35280 | 102.26 |
|  | 150 | 120 | 585 | 83.57% | 11 | 1.57% | 94 | 13.43% | 98.57% | 72 | 10.29% | 420 | 271 | 48780 | 116.14 |
|  | 200 | 5 | 45 | 6.43% | 539 | 77.00% | 66 | 9.43% | 92.86% | 0 | 0.00% | 405 | 206 | 37080 | 91.56 |
|  | 200 | 10 | 213 | 30.43% | 295 | 42.14% | 151 | 21.57% | 94.14% | 1 | 0.14% | 410 | 211 | 37980 | 92.63 |
|  | 200 | 20 | 498 | 71.14% | 37 | 5.29% | 123 | 17.57% | 94.00% | 3 | 0.43% | 420 | 221 | 39780 | 94.71 |
|  | 200 | 45 | 570 | 81.43% | 1 | 0.14% | 116 | 16.57% | 98.14% | 24 | 3.43% | 445 | 246 | 44280 | 99.51 |
|  | 200 | 120 | 692 | 98.86% | 0 | 0.00% | 0 | 0.00% | 98.86% | 78 | 11.14% | 520 | 321 | 57780 | 111.12 |
|  | 200 | 180 | 616 | 88.00% | 8 | 1.14% | 65 | 9.29% | 98.43% | 71 | 10.14% | 580 | 381 | 68580 | 118.24 |
|  | 420 | 120 | 689 | 98.43% | 0 | 0.00% | 0 | 0.00% | 98.43% | 30 | 4.29% | 960 | 541 | 97380 | 101.44 |
| **150** | 150 | 75 | 477 | 68.14% | 6 | 0.86% | 209 | 29.86% | 98.86% | 97 | 13.86% | 375 | 226 | 67800 | 180.80 |
|  | 220 | 75 | 576 | 82.29% | 1 | 0.14% | 114 | 16.29% | 98.71% | 57 | 8.14% | 515 | 296 | 88800 | 172.43 |
|  | 220 | 200 | 691 | 98.71% | 0 | 0.00% | 0 | 0.00% | 98.71% | 68 | 9.71% | 640 | 421 | 126300 | 197.34 |
|  | 350 | 5 | 50 | 7.14% | 554 | 79.14% | 67 | 9.57% | 95.86% | 0 | 0.00% | 705 | 356 | 106800 | 151.49 |
|  | 350 | 10 | 69 | 9.86% | 447 | 63.86% | 158 | 22.57% | 96.29% | 0 | 0.00% | 710 | 361 | 108300 | 152.54 |
|  | 350 | 20 | 513 | 73.29% | 37 | 5.29% | 126 | 18.00% | 96.57% | 2 | 0.29% | 720 | 371 | 111300 | 154.58 |
|  | 350 | 75 | 474 | 67.71% | 6 | 0.86% | 212 | 30.29% | 98.86% | 42 | 6.00% | 775 | 426 | 127800 | 164.90 |
|  | 350 | 200 | 691 | 98.71% | 0 | 0.00% | 0 | 0.00% | 98.71% | 43 | 6.14% | 900 | 551 | 165300 | 183.67 |
|  | 350 | 300 | 678 | 96.86% | 10 | 1.43% | 2 | 0.29% | 98.57% | 62 | 8.86% | 1000 | 651 | 195300 | 195.30 |
|  | 530 | 200 | 691 | 98.71% | 0 | 0.00% | 0 | 0.00% | 98.71% | 35 | 5.00% | 1260 | 731 | 219300 | 174.05 |

Note: We simulated 700 virus insertion events in each row.

Correct: Distance between simulated and found point is within 10 bp range.

True Positive: Both human split site and virus split site are correct. False Positive: Human split site is wrong.

HumOnly: Human split site is right but virus split site is wrong.

VirNA: Human split site is right but virus split site is not found.

HumCalled: Sum of left (all listed except for FP), which is the called rate on human genome.

Cnt: Count

PE: Paired-end read

## Figure S1. The performance of bs-virus-finder in various length of virus integration using PE50.

We simulated 100 virus insertion events for each dot with.

TP for True Positive: Both human split site and virus split site are correct.

FP for False Positive: Human split site is wrong.

HumOnly: Human split site is right but virus split site is wrong.

VirNA: Human split site is right but virus split site is not found.

Correct is defined as: Distance between simulated and found point is within 10 bp range.

## Figure S2. The performance of bs-virus-finder in various length of virus integration using PE90.

## Figure S3. The performance of bs-virus-finder in various length of virus integration using PE150.


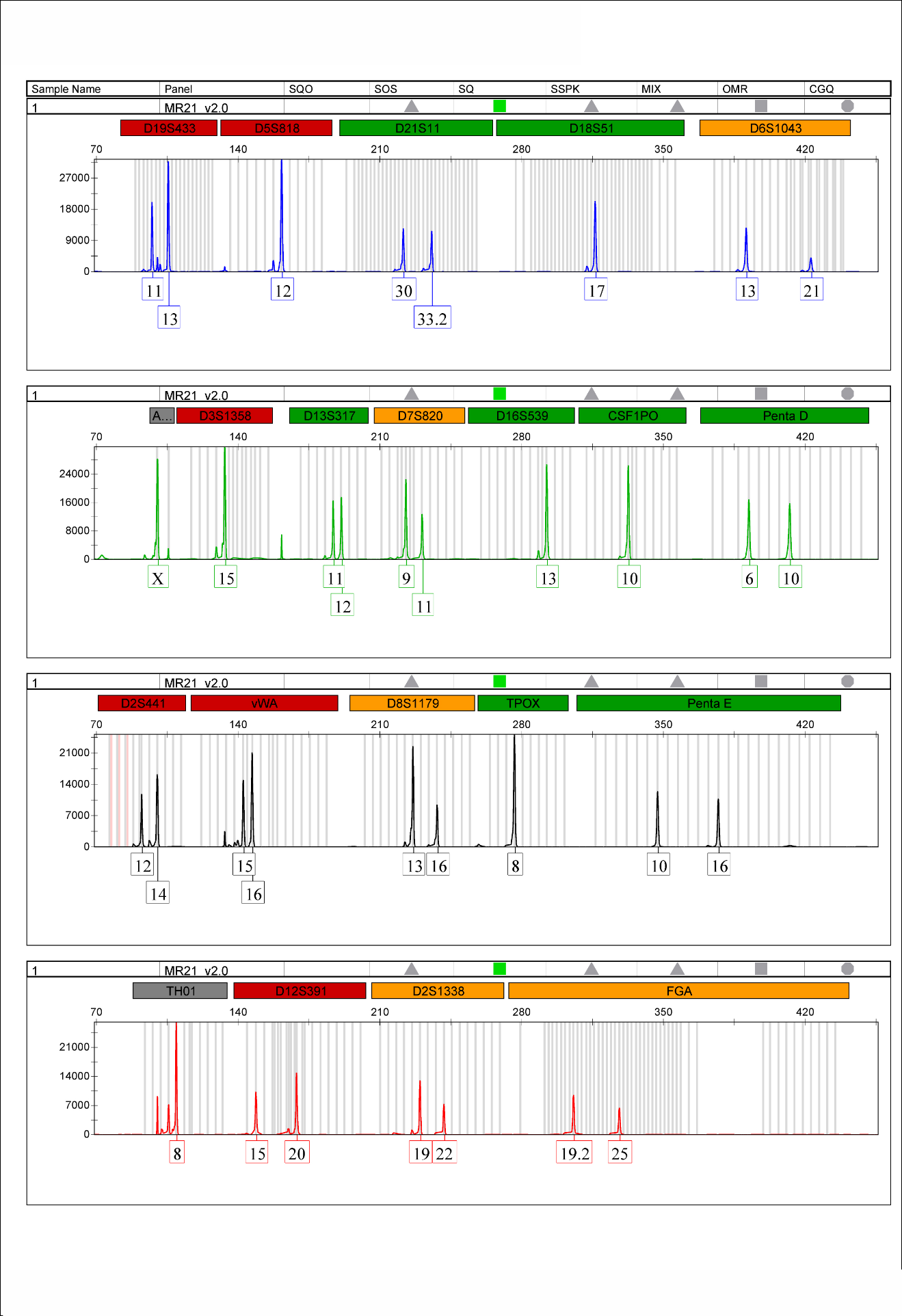


## Figure S4. The diagram of STR for PLC/PRF/5 cell line.

The PLC/PRF/5 hepatocellular carcinoma cell lines were from American Type Culture Collection (ATCC, Manassas, VA). Genomic DNA was isolated by chelex-100 (Promega Biotech Co., Ltd). The samples were amplified using the Microreader^TM^ 23 ID system (Suzhou Microread Genetics, Suzhou, Jiangsu, China), PCR amplification was carried out on a veriti cycler (Applied Biosystems, USA) following the manufacturer’s instructions, and amplified products were separated by capillary electrophoresis on a 3500 DNA Genetic Analyzer (Applied Biosystems, USA). Electrophoresis results were analyzed using GeneMapper ID-X 1.4 software (Applied Biosystems). The genotypes are as follows: Amelogenin: X; CSF1PO: 10; D13S317: 11,12; D16S539: 13; D5S818: 12; D7S820: 9,11; THO1: 8; TPOX: 8; vWA: 15,16. All the genotypes of these STR loci were consistent with the standard genotyps which were supposed to.
