# Supplementary material for: BS-virus-finder: virus integration calling using bisulfite sequencing data
Source: Gigascience. 2017 Dec 18;7(1):1–7. doi: 10.1093/gigascience/gix123 (PMC5788064; doi:10.1093/gigascience/gix123)
Supplement: Response_to_Reviewer_Comments_Original_Submission.pdf [file gix123_response_to_reviewer_comments_original_submission.pdf]

Author's Response To Reviewer Comments

Reviewer #1: Major Concerns
1. Why did the authors choose whole-genome bisulfite sequencing (WGBS) data for identification of the virus integration loci? There are many established tools or pipelines for detection of viral integration loci based on whole-genome sequencing (WGS) and/or RNA-seq. What is the rationale for developing a method using WGBS instead of improving upon the methods for WGS or RNA-seq for virus integration detection? Authors provide a reference, saying, "A recent clinical study showed that DNA methylation is associated with viral integration", but the work of Larsson GL et al, 2014 was not performed on whole-genome methylation data and hardly could be used as a rationale for using WGBS for the virus detection.

Thank you for your comments. There is no existing method for analysis of virus integration by using WGBS data unless additional relative WGS and RNA-seq data is available, thus requiring more human and financial resources. Regarding this, our software tool solved this problem by finding virus integration directly from WGBS data, therefore enabling efficiently and deeply mining data. We cite the work of Larsson GL to show that there is a relationship between virus integration and DNA methylation, and not as a rationale for using WGBS.

2. Authors listed several tools for the alignment of WGBS data on page 5 lines 18-20. Why did the authors use BWA-meth instead of another published tool? BWA-meth is not published in a peer-reviewed journal, whereas other aligners such as Bismark are, so authors should provide a rationale for choosing this aligner.

Soft clipping information was required when we initiate our search for the virus-integrated sites. However, there is no such function that can be used to provide this information by using the previous software (neither bsmap nor bismark) for sequence comparison. Therefore, we chose bwameth and bsseeker2 for the sequence comparison and we found BWA-meth showed the best. We finally applied BWA-meth as the software for comparision.

3. Simulation should be described/performed better. For example, what bisulfite conversion rate for cytosines in CG-context was used in this simulation? There are tools for bisulfite read simulation, for example, SHERMAN which allows users to simulate bisulfite reads with varying bisulfite conversion rate.

We applied SHERMAN to randomly model 100 break points with 20% conversation rate. Out of 94 break points, 89 are correct, 5 are false positive., This result is similar with our preliminary modeling result.

4. It is not clear from the manuscript whether authors tried to find real data for testing BS-virus-finder. The authors should include results using real WGBS data in addition to simulated data. If there is no such data, then there is no reason to develop a method for it.

We performed WGS and WGBS for PLC/PRF/5 cell line and anaylizer the data. The result is showed in Table 1.

5. Performance of BS-virus-finder should be compared with performance of the existing tools/pipelines for detection viral integration on WGS and RNA-seq (for example VirusSeq). Authors could remove BS-conversion from their simulated data and use them for running VirusSeq or other established pipeline for virus detection. But using real data would be better.

We performed WGS and WGBS for PLC/PRF/5 cell line and anaylizer the data. The result is showed in Table 1.

6. The section "Method for calling virus integration" is written from the point of view that authors know which reads contain junctions. This is could be true for simulated data but not for real data. It raises many questions through the Methods section - how will it work on real data? Probably authors should re-write text in the context of working with real (or proper simulated) reads, when users do not know which reads contain junctions.

We performed WGC and WGBS for PLC/PRF/5 cell line and detected by BSVF, respectively. The result is showed in Table 1.

7. In the sentence "We used Bwa-meth to align junction reads and mark the shorter junction parts as soft-clip" why are shorter junction parts marked as soft-clip? How will you know which parts of real reads are short and should be marked as soft-clip?

Thank you for your comments, we edited the text to reducing the confusing points you mentioned.

8. There is a lack of details about filtering the alignment results (page 7 lines 7-8): sequencing quality, mapping quality and mismatch rates should be described better with specific parameters for every step.

Thank you for your comments, we revised the text based on your suggestion.

9. There is lack of details regarding clustering procedure of reads surrounding or containing breakpoints. The clustering procedure (cluster extension) could be supported by a scheme/figure for better understanding. Also, which reads will you cluster in real data when you do not know which of them contain breakpoints? Reads which are not aligned to the reference human genome? This should be described in the text.

Thank you for your comments, we edited the text based on your suggestion.

10. Section Assembling could be accompanied by a better scheme/figure or more text for the author's restore algorithm. Figure 2 does not clearly explain how the restore algorithm is restoring the bisulfite-altered sequence to the original and more details are needed. For example, which strain on Figure 2 is original and which is restored.

Thank you for your comments, we revised this part to make the method clearer.

11. Also, are there any studies where such an approach for assembling (as author's restore

algorithm) was previously used? References should be provided or it should be mentioned if it is completely novel approach.

Thank you for your comments, we edited the text to make the new approach clearer.

12. Last part of the "Methods" suggests alignment of unmapped to the human reference genome reads to the viral reference sequence. In real data when you do not know what types of viruses are contained/integrated in the analyzed sample which viral references should the user use? Should it be all known viral reference sequences? Or should the user perform an initial analysis for identification of virus(es) in the sample and then use this pipeline only for detection of breakpoints (as in VirusSeq)?

We performed WGS and WGBS for PLC/PRF/5 cell line and anaylizer the data. The result is showed in Table 1.

13. Figure 3 needs more description in text of what exactly it shows, and a clearer explanation in the legend. I do not see how Figure 3 demonstrates the extraction of the virus fragment location from the alignment result.

Thank you for your comments, we edited the text based on your suggestion.

Minor Concerns

1. Manuscript pages and formulas must be numbered.

Thank you for your comments, we numbered the text based on your suggestion.

2. There are discrepancies in the text regarding what chromosome was used for simulation: chr 18 on page 6 line 6 and chr 1 on page 10 line 13. In the section "Data description in silico" authors mentioned that simulation of breakpoints was performed only on PE reads (90 bp), but in Table 1 and in Discussion they are mentioned simulation of PE 50, 90, 150. Authors should coordinate through all sections of the article - what and how they performed analysis and simulation in this study.

Thank you for your comments, we edited the text based on your suggestion.

3. In the section Assembling "Q" should be defined in second formula (page 8, line 12).

Thank you for your comments, we edited the text based on your suggestion.

4. In "Discussion" (page 9, line 12) a reference should be provided for the statement "Virus usually integrates into regions that homologous to both human and virus (micro-homologous)".

Thank you for your comments, we edited the text based on your suggestion.

5. On page 9 lines 14-15 authors claim "The accuracy of predicted breakpoints can reach over 70%" and then on page 10, line 1 "Bs-virus-finder is capable to find more than 80% of virus integration with the accuracy more than 90%". Should be consistent in description of simulation's results.

Thank you for your comments, we revised the text based on your suggestion. Particularly, as the result showed in Table S4, for input sequence that the length around 50bp, BS-virus-finder is capable to find the virus integration with the accuracy more than 70%; for the input sequence between 90bp and 150bp, BS-virus-finder is capable to find the virus integration with the accuracy more than 90%.

6. There are many English grammar errors through the text which should be corrected. For example, stimulated instead of simulated. Also, in sentence "Generally, however, bwa-meth [13] performed very well. It indicated virus breakpoints might be hardly found by our BS virus finder" if breakpoints could be hardly found, why was this manuscript written?

We have deleted this confusing describing.

7. Paragraph on page 6, lines 15-19 not suits to Result section and should be moved to Introduction, for example.

Thank you for your comments, we revised the text based on your suggestion.

Reviewer #2: The study presented by Gao and colleagues discusses a software, BS-virus-finder, which allows the detection of viral integration breakpoints in human genomes using bisulfite sequencing data. Importantly, this appears to be the first software which allows the detection of viral integration breakpoints from bisulfite sequencing data.

1) Introduction: Define the abbreviations 'SNP', 'DMR' and 'ASM'.

Thank you for your comments, we edited the text based on your suggestion.

2) Introduction: Abbreviations need to be harmonised: The abbreviation for 'whole-genome bisulfite sequencing' is given as 'Bis-seq' and 'WGBS'.

Thank you for your comments, we edited the text based on your suggestion.

3) Introduction: The software SMAP appears to be referenced as reference [11] as well as reference [1].

Thank you for your comments, we edited the text based on your suggestion.

4) Data description in silico: I don't understand the following sentences: 'Generally, however, bwa-meth [13] performed very well. It indicated virus breakpoints might be hardly found by our BS virus finder.' - Does this mean that the performance of the bwa-meth software alone is superior to the presented BS-virus-finder software which is based on bwa-meth? Please clarify.

Thank you for your comments, we edited the text based on your suggestion. We deleted the confusing description.

5) The authors should provide a table where they compare the BS-virus-finder software with other software used for the detection of viral integration breakpoints, such as VirusFinder (PMID: 23717618), VERSE (PMID: 25699093), Virus-Clip (PMID: 26087185), Vy-PER (PMID: 26166306), Seeksv (PMID: 27634948) or any other software of relevance.

We performed WGS and WGBS for PLC/PRF/5 cell line. The result was analyzed by Vy-per, virus-clip(REF) and Virus Finder2, respectively. These results were compared with WGBS result analyzed by BSVF. The comparison of the result is showed in Table 1.

6) It would be good if the authors could provide an example/examples where they show the performance of the BS-virus-finder on 'real' datasets (perhaps datasets which have been analysed by using other software tools?).

We performed WGS and WGBS for PLC/PRF/5 cell line. The result were analyzed by Vy-per, virus-clip and Virus Finder2, respectively. These results were compared with WGBS result analyzed by BSVF. The comparison of the result is showed in Table 1.

7) Figure 2/Legend figure 2: The 'G' in a 'CG' shows for the 'Crick' strand the 'm' in subscript to indicate that this is a methylation-modified base. However, this is confusing as it leaves the impression that the 'G' is methylated instead of the corresponding 'C'.

Methylation actually occurs at C, however when C in Crick strand transformed into T, at its reverse complementary strand it is G transformed into A. Therefore, Gm equals Cm at Crick strand.
