# Supplementary material for: BS-virus-finder: virus integration calling using bisulfite sequencing data
Source: Gigascience. 2017 Dec 18;7(1):1–7. doi: 10.1093/gigascience/gix123 (PMC5788064; doi:10.1093/gigascience/gix123)
Supplement: Response_to_Reviewer_Comments_Revision_2.pdf [file gix123_response_to_reviewer_comments_revision_2.pdf]

## Author's Response To Reviewer Comments

GIGA-D-17-00032R2
BS-virus-finder: virus integration calling using bisulfite-sequencing data
Shengjie Gao; Xuesong Hu; Changduo Gao; Kai Xiong; Fengping Xu; Xiao Zhao; Haixiao Chen; Shancen Zhao; Mengyao Wang; Dongke Fu; Xiaohui Zhao; Jie Bai; Likai Mao; Bo Li; Song Wu; Jian Wang; Shengbin Li; Huanming Yang; Lars Bolund; Christian Pedersen
GigaScience

Dear Christian,

Please refer to my previous email from my personal account and the attached word doc. This email is just to convince Editorial Manager to let you upload your final version via the system.

Once you have made the necessary corrections, please submit a revised manuscript online at:

http://giga.edmgr.com/

If you have forgotten your username or password please use the "Send Login Details" link to get your login information. For security reasons, your password will be reset.

Thank you sir! I have completed the revision and submitted the final version.
