# Supplementary material for: BS-virus-finder: virus integration calling using bisulfite sequencing data
Source: Gigascience. 2017 Dec 18;7(1):1–7. doi: 10.1093/gigascience/gix123 (PMC5788064; doi:10.1093/gigascience/gix123)
Supplement: Reviewer_1_Report_(Original_Submission) -- Lada Koneva — 07 Mar 2017 Reviewed [file gix123_reviewer_1_report_(original_submission).pdf]

Reviewer Report

Title: BS-virus-finder: virus integration calling using bisulfite-sequencing data

Version: Original Submission **Date:** 07 Mar 2017

Reviewer name: Lada Koneva

Reviewer Comments to Author:

Major Concerns

1. Why did the authors choose whole-genome bisulfite sequencing (WGBS) data for identification of the virus integration loci? There are many established tools or pipelines for detection of viral integration loci based on whole-genome sequencing (WGS) and/or RNA-seq. What is the rationale for developing a method using WGBS instead of improving upon the methods for WGS or RNA-seq for virus integration detection? Authors provide a reference, saying, "A recent clinical study showed that DNA methylation is associated with viral integration", but the work of Larsson GL et al, 2014 was not performed on whole-genome methylation data and hardly could be used as a rationale for using WGBS for the virus detection.
2. Authors listed several tools for the alignment of WGBS data on page 5 lines 18-20. Why did the authors use BWA-meth instead of another published tool? BWA-meth is not published in a peer-reviewed journal, whereas other aligners such as Bismark are, so authors should provide a rationale for choosing this aligner.
3. Simulation should be described/performed better. For example, what bisulfite conversion rate for cytosines in CG-context was used in this simulation? There are tools for bisulfite read simulation, for example, SHERMAN which allows users to simulate bisulfite reads with varying bisulfite conversion rate.
4. It is not clear from the manuscript whether authors tried to find real data for testing BS-virus-finder. The authors should include results using real WGBS data in addition to simulated data. If there is no such data, then there is no reason to develop a method for it.
5. Performance of BS-virus-finder should be compared with performance of the existing tools/pipelines for detection viral integration on WGS and RNA-seq (for example VirusSeq). Authors could remove BS-conversion from their simulated data and use them for running VirusSeq or other established pipeline for virus detection. But using real data would be better.
6. The section "Method for calling virus integration" is written from the point of view that authors know which reads contain junctions. This is could be true for simulated data but not for real data. It raises many questions through the Methods section - how will it work on real data? Probably authors should re-write text in the context of working with real (or proper simulated) reads, when users do not know which reads contain junctions.
7. In the sentence "We used Bwa-meth to align junction reads and mark the shorter junction parts as soft-clip" why are shorter junction parts marked as soft-clip? How will you know which parts of real reads are short and should be marked as soft-clip?
8. There is a lack of details about filtering the alignment results (page 7 lines 7-8): sequencing quality, mapping quality and mismatch rates should be described better with specific parameters for every step.

9. There is lack of details regarding clustering procedure of reads surrounding or containing breakpoints. The clustering procedure (cluster extension) could be supported by a scheme/figure for better understanding. Also, which reads will you cluster in real data when you do not know which of them contain breakpoints? Reads which are not aligned to the reference human genome? This should be described in the text.
10. Section Assembling could be accompanied by a better scheme/figure or more text for the author's restore algorithm. Figure 2 does not clearly explain how the restore algorithm is restoring the bisulfite-altered sequence to the original and more details are needed. For example, which strain on Figure 2 is original and which is restored.
11. Also, are there any studies where such an approach for assembling (as author's restore algorithm) was previously used? References should be provided or it should be mentioned if it is completely novel approach.
12. Last part of the "Methods" suggests alignment of unmapped to the human reference genome reads to the viral reference sequence. In real data when you do not know what types of viruses are contained/integrated in the analyzed sample which viral references should the user use? Should it be all known viral reference sequences? Or should the user perform an initial analysis for identification of virus(es) in the sample and then use this pipeline only for detection of breakpoints (as in VirusSeq)?
13. Figure 3 needs more description in text of what exactly it shows, and a clearer explanation in the legend. I do not see how Figure 3 demonstrates the extraction of the virus fragment location from the alignment result.

Minor Concerns
1. Manuscript pages and formulas must be numbered.
2. There are discrepancies in the text regarding what chromosome was used for simulation: chr 18 on page 6 line 6 and chr 1 on page 10 line 13. In the section "Data description in silico" authors mentioned that simulation of breakpoints was performed only on PE reads (90 bp), but in Table 1 and in Discussion they are mentioned simulation of PE 50, 90, 150. Authors should coordinate through all sections of the article - what and how they performed analysis and simulation in this study.
3. In the section Assembling "Q" should be defined in second formula (page 8, line 12).
4. In "Discussion" (page 9, line 12) a reference should be provided for the statement "Virus usually integrates into regions that homologous to both human and virus (micro-homologous)".
5. On page 9 lines 14-15 authors claim "The accuracy of predicted breakpoints can reach over 70%" and then on page 10, line 1 "Bs-virus-finder is capable to find more than 80% of virus integration with the accuracy more than 90%". Should be consistent in description of simulation's results.
6. There are many English grammar errors through the text which should be corrected. For example, stimulated instead of simulated. Also, in sentence "Generally, however, bwa-meth [13] performed very well. It indicated virus breakpoints might be hardly found by our BS virus finder" if breakpoints could be hardly found, why was this manuscript written?
7. Paragraph on page 6, lines 15-19 not suits to Result section and should be moved to Introduction, for example.

Level of Interest

Please indicate how interesting you found the manuscript: An article of limited interest

Quality of Written English

Please indicate the quality of language in the manuscript: Not suitable for publication unless extensively edited

Declaration of Competing Interests

Please complete a declaration of competing interests, considering the following questions:

- Have you in the past five years received reimbursements, fees, funding, or salary from an organisation that may in any way gain or lose financially from the publication of this manuscript, either now or in the future?
- Do you hold any stocks or shares in an organisation that may in any way gain or lose financially from the publication of this manuscript, either now or in the future?
- Do you hold or are you currently applying for any patents relating to the content of the manuscript?
- Have you received reimbursements, fees, funding, or salary from an organization that holds or has applied for patents relating to the content of the manuscript?
- Do you have any other financial competing interests?
- Do you have any non-financial competing interests in relation to this paper?

If you can answer no to all of the above, write 'I declare that I have no competing interests' below. If your reply is yes to any, please give details below.

I declare that I have no competing interests.

I agree to the open peer review policy of the journal. I understand that my name will be included on my report to the authors and, if the manuscript is accepted for publication, my named report including any attachments I upload will be posted on the website along with the authors' responses. I agree for my report to be made available under an Open Access Creative Commons CC-BY license (http://creativecommons.org/licenses/by/4.0/). I understand that any comments which I do not wish to be included in my named report can be included as confidential comments to the editors, which will not be published.

I agree to the open peer review policy of the journal
