# Supplementary material for: BS-virus-finder: virus integration calling using bisulfite sequencing data
Source: Gigascience. 2017 Dec 18;7(1):1–7. doi: 10.1093/gigascience/gix123 (PMC5788064; doi:10.1093/gigascience/gix123)
Supplement: Reviewer_1_Report_(Revision_1) -- Lada Koneva — 22 Sep 2017 Reviewed [file gix123_reviewer_1_report_(revision_1).pdf]

Reviewer Report

Title: BS-virus-finder: virus integration calling using bisulfite-sequencing data

Version: Revision 1 **Date:** 22 Sep 2017

Reviewer name: Lada Koneva

Reviewer Comments to Author:

Re-review on manuscript
BS-virus-finder: virus integration calling using bisulfite-sequencing data
Shengjie Gao, Ph.D; Xuesong Hu; Changduo Gao; Kai Xiong; Fengping Xu; Xiao Zhao; Haixiao Chen; Shancen Zhao; Mengyao Wang; Dongke Fu; Xiaohui Zhao; Jie Bai; Bo Li; Song Wu; Shengbin Li; Huanming Yang; Lars Bolund; Christian Pedersen.
by Lada A Koneva, PhD

Introduction
The reviewed manuscript (BS-virus-finder: virus integration calling using bisulfite-sequencing data) describes developed software to detect viral integration breakpoints in whole-genome bisulfite-sequencing data (WGBS).
The manuscript was improved according to my suggested concerns and questions, but there are still some questions.

Questions
As a consequence of choosing HBV for simulation and PLC/PRF/5 hepatocellular carcinoma cell lines (which harboring HBV) as a real data, there is still unanswered my question about how this algorithm will work in case of HPV integration. There are about 170 types of HPV, with high commonality between different types, and about 15 types are carcinogenic. How authors suggest creating a "hybrid reference that contains both human genome and virus sequences" in case of HPV? Which viral types could be chosen for alignment of assembled clipping regions in case of HPV contamination? Probably this algorithm could not accurately predict which type(s) of HPV contaminates the sample. It would be useful if authors could provide their thoughts if this algorithm also will work in case of virus like HPV or this should be restricted to analysis of HBV integration only.

How many reads were simulated in each simulation scenario? Did the authors vary the sequencing depth in the simulations?

Minor
1. Section "Description in silico and real data": authors mentioned that simulated reads were selected from chr 18 of GRCh38 and then next sentence "Input fragments were selected from chr 18 in the GRCh37 assembly (hg19) of the human genome". Please, correct this if it's your typo, or clarify why different versions of reference genome were used for simulation.

2. Also they are still using the word "stimulated" instead of "simulated". And in the section "Availability of supporting data" the chromosome 1 was mentioned as chosen for simulation. Please correct it. Also typo on page 14-15: "We stimulated three kinds of reads, PE50, 90, and 50" should be 150.
3. Legend to Figure 1: The sentence "Methylation sites were showed as read bases" do you mean "red" bases?
4. The information for which virus was used for the simulation of the viral integration is mentioned only at the end of the manuscript (Availability of supporting data). Please provide this information in section "Description in silico and real data".

Level of Interest

Please indicate how interesting you found the manuscript: An article of importance in its field

Quality of Written English

Please indicate the quality of language in the manuscript: Needs some language corrections before being published

Declaration of Competing Interests

Please complete a declaration of competing interests, considering the following questions:

- Have you in the past five years received reimbursements, fees, funding, or salary from an organisation that may in any way gain or lose financially from the publication of this manuscript, either now or in the future?
- Do you hold any stocks or shares in an organisation that may in any way gain or lose financially from the publication of this manuscript, either now or in the future?
- Do you hold or are you currently applying for any patents relating to the content of the manuscript?
- Have you received reimbursements, fees, funding, or salary from an organization that holds or has applied for patents relating to the content of the manuscript?
- Do you have any other financial competing interests?
- Do you have any non-financial competing interests in relation to this paper?

If you can answer no to all of the above, write 'I declare that I have no competing interests' below. If your reply is yes to any, please give details below.

I declare that I have no competing interests

I agree to the open peer review policy of the journal. I understand that my name will be included on my report to the authors and, if the manuscript is accepted for publication, my named report including any attachments I upload will be posted on the website along with the authors' responses. I agree for my report to be made available under an Open Access Creative Commons CC-BY license (http://creativecommons.org/licenses/by/4.0/). I understand that any comments which I do not wish to be included in my named report can be included as confidential comments to the editors, which will not be published.

I agree to the open peer review policy of the journal
