# Supplementary material for: BS-virus-finder: virus integration calling using bisulfite sequencing data
Source: Gigascience. 2017 Dec 18;7(1):1–7. doi: 10.1093/gigascience/gix123 (PMC5788064; doi:10.1093/gigascience/gix123)
Supplement: Reviewer_2_Report_(Original_Submission) -- Thomas Mikeska — 07 Mar 2017 Reviewed [file gix123_reviewer_2_report_(original_submission).pdf]

Reviewer Report

Title: BS-virus-finder: virus integration calling using bisulfite-sequencing data

Version: Original Submission **Date:** 07 Mar 2017

Reviewer name: Thomas Mikeska

Reviewer Comments to Author:

The study presented by Gao and colleagues discusses a software, BS-virus-finder, which allows the detection of viral integration breakpoints in human genomes using bisulfite sequencing data. Importantly, this appears to be the first software which allows the detection of viral integration breakpoints from bisulfite sequencing data.

1) Introduction: Define the abbreviations 'SNP', 'DMR' and 'ASM'.

2) Introduction: Abbreviations need to be harmonised: The abbreviation for 'whole-genome bisulfite sequencing' is given as 'Bis-seq' and 'WGBS'.

3) Introduction: The software SMAP appears to be referenced as reference [11] as well as reference [1].

4) Data description in silico: I don't understand the following sentences: 'Generally, however, bwa-meth [13] performed very well. It indicated virus breakpoints might be hardly found by our BS virus finder.' - Does this mean that the performance of the bwa-meth software alone is superior to the presented BS-virus-finder software which is based on bwa-meth? Please clarify.

5) The authors should provide a table where they compare the BS-virus-finder software with other software used for the detection of viral integration breakpoints, such as VirusFinder (PMID: 23717618), VERSE (PMID: 25699093), Virus-Clip (PMID: 26087185), Vy-PER (PMID: 26166306), Seeksv (PMID: 27634948) or any other software of relevance.

6) It would be good if the authors could provide an example/examples where they show the performance of the BS-virus-finder on 'real' datasets (perhaps datasets which have been analysed by using other software tools?).

7) Figure 2/Legend figure 2: The 'G' in a 'CG' shows for the 'Crick' strand the 'm' in subscript to indicate that this is a methylation-modified base. However, this is confusing as it leaves the impression that the 'G' is methylated instead of the corresponding 'C'.

Level of Interest

Please indicate how interesting you found the manuscript: An article whose findings are important to those with closely related research interests

Quality of Written English

Please indicate the quality of language in the manuscript: Needs some language corrections before being published

Declaration of Competing Interests

Please complete a declaration of competing interests, considering the following questions:

- Have you in the past five years received reimbursements, fees, funding, or salary from an organisation that may in any way gain or lose financially from the publication of this manuscript, either now or in the future?
- Do you hold any stocks or shares in an organisation that may in any way gain or lose financially from the publication of this manuscript, either now or in the future?
- Do you hold or are you currently applying for any patents relating to the content of the manuscript?
- Have you received reimbursements, fees, funding, or salary from an organization that holds or has applied for patents relating to the content of the manuscript?
- Do you have any other financial competing interests?
- Do you have any non-financial competing interests in relation to this paper?

If you can answer no to all of the above, write 'I declare that I have no competing interests' below. If your reply is yes to any, please give details below.

I declare that I have no competing interests

I agree to the open peer review policy of the journal. I understand that my name will be included on my report to the authors and, if the manuscript is accepted for publication, my named report including any attachments I upload will be posted on the website along with the authors' responses. I agree for my report to be made available under an Open Access Creative Commons CC-BY license (http://creativecommons.org/licenses/by/4.0/). I understand that any comments which I do not wish to be included in my named report can be included as confidential comments to the editors, which will not be published.

I agree to the open peer review policy of the journal
